# Conformational asymmetry of human immunodeficiency virus‐1 protease flaps increases along the darunavir drug resistance pathway

**DOI:** 10.1002/pro.70736

**Published:** 2026-08-05

**Authors:** Michel Guerrero, Roman Zadorozhnyi, Guowu Lin, Caitlin M. Quinn, Guillermo Calero, Nese Kurt‐Yilmaz, Celia A. Schiffer, Tatyana Polenova, Angela M. Gronenborn, Rieko Ishima

**Affiliations:** ^1^ Department of Structural Biology University of Pittsburgh School of Medicine Pittsburgh Pennsylvania USA; ^2^ Department of Chemistry and Biochemistry University of Delaware Newark Delaware USA; ^3^ Pittsburgh Center for HIV Protein Interactions University of Pittsburgh School of Medicine Pittsburgh Pennsylvania USA; ^4^ Department of Biochemistry and Molecular Biotechnology University of Massachusetts Chan Medical School Worcester Massachusetts USA

**Keywords:** dynamics, fluorine‐19 NMR, HIV‐1 protease, NMR, protein dynamics, relaxation, structure

## Abstract

Under selective drug pressure, the structure and dynamics of HIV‐1 protease (PR) evolve to confer resistance to the drug. Here, we report on the conformational changes in PR flaps for drug‐resistance mutations using ^19^F nuclear magnetic resonance (NMR) spectroscopy. We prepared wild‐type (wt) PR and its three mutant variants containing 4, 10, or 11 mutations, namely 4Mut, 10Mut, and 11Mut, that were previously found in a viral passage study against darunavir (DRV). The proteins were uniformly labeled with ^15^N and with ^19^F amino‐acid specifically in the 5‐indole positions of two conserved tryptophan residues, W6 and W42, which are located near the dimer interface and at the edge of the flap region of PR, respectively. In solution, ^19^F NMR spectra of these proteins in the inhibitor‐free forms showed a downfield shift of the W42 resonance as the number of mutations increased. In the DRV‐bound form, the W42 resonance again shifted with increasing number of mutations and, importantly, split into two peaks, reflecting different local electronic environments of the two flaps of the dimer. Solid‐state ^19^F magic angle spinning (MAS) NMR spectra of wt‐PR and 10Mut also exhibited two W42 resonances in DRV‐bound crystalline forms. These observations indicate that drug‐resistance mutations increase the asymmetry between the two flaps in the PR‐inhibitor interaction in the inhibitor‐bound forms.

## 1 INTRODUCTION

**FIGURE 1:**
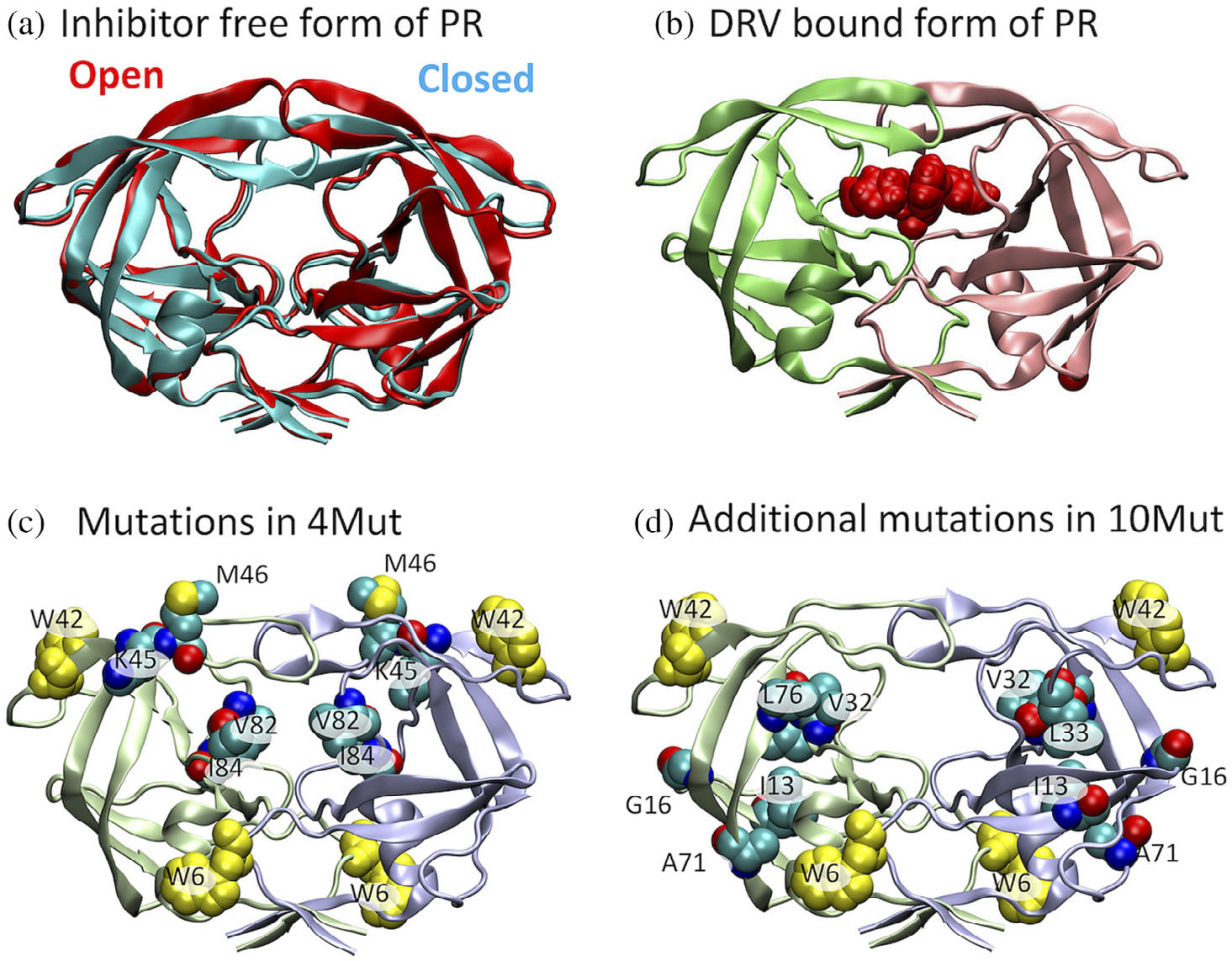
Superposition of HIV‐1 wt‐PR structures in ribbon presentation for (a) inhibitor‐free and (b) DRV‐bound forms, and (c) mutation sites (in ball representation) for 4Mut (K45I, M46I, V82F, and I84V) and (d) additional mutation sites for 10Mut (I13V, G16E, V32I, L33F, A71V, and L76V). Note, 11Mut has one additional mutation, I54L, on 10Mut. Panel (a) was generated with two PR structures, one is closed (cyan color ribbons, PDB code 4EJL; Tiefenbrunn et al., 2013), and the other is more open (red color ribbons, PDB code 1HHP; Spinelli et al., 1991), to give a flavor of dynamics of the flap region. Panels (b–d) were generated using PDB 6DGX (Henes et al., 2019). In panel (b), two chains of HIV‐PR are shown by green and pink, with DRV (red color) between the two. In panels (c) and (d), mutation sites and Trp residues are shown in ball representation using either the standard CPK colors or yellow color, respectively.

Human immunodeficiency virus‐1 (HIV‐1) protease (PR) is an enzyme essential for HIV‐1 replication (Coffin et al., 1997; Kaplan et al., 1993; Kohl et al., 1988; Krausslich, 1991; Oroszlan & Luftig, 1990; Seelmeier et al., 1988) and has long been a target for drug development (Erickson & Burt, 1996; Galiano et al., 2009; Ghosh et al., 2008; Quinones‐Mateu et al., 2008; Surleraux et al., 2005; Wlodawer & Erickson, 1993). The mature HIV‐1 PR is a homodimeric aspartyl protease, with each subunit comprising 99 amino acids that together form an active site with two aspartic acid residues, one from each of the two subunits (Lapatto et al., 1989; Navia et al., 1989; Wlodawer et al., 1989). The flap region of the PR (residues 43–58) is known to be dynamic, exhibiting an ensemble of open and closed conformations in solution (Figure 1a); the closed conformation predominates upon binding to competitive inhibitors (Figure 1b). For characterizing PR flap dynamics, nuclear magnetic resonance (NMR) spectroscopy has made significant contributions (Blackburn et al., 2009; Freedberg et al., 2002; Galiano et al., 2009; Ishima et al., 1998; Ishima et al., 1999; Ishima & Louis, 2008; Louis et al., 2007; Nicholson et al., 1995; Roche et al., 2015), and findings from NMR studies have been supported by a large number of PR crystal‐structure‐based computational studies that helped to provide insight into the motion of PR in the free and inhibitor‐bound forms (Chetty et al., 2016; Collins et al., 1995; Deng et al., 2011; Ding et al., 2008; Gardner & Abrams, 2019; Harrison & Weber, 1994; Harte et al., 1990; Hou & Yu, 2007; Karthik & Senapati, 2011; Leidner et al., 2021; Paulsen et al., 2017; Perryman et al., 2006; Ragland et al., 2017; Shen et al., 2015; Silva et al., 1996; Toth & Borics, 2006; Tzoupis et al., 2014; Ung et al., 2014; Venkatachalam et al., 2025; Wang & Zheng, 2021; York et al., 1993).

Differences in the structure and dynamics of PR in the presence of mutations that impart multidrug resistance also have been well studied: most mutations generally increase flap mobility in inhibitor‐free forms of the protein, reduce packing at the inhibitor‐PR interface compared to the wild‐type protein, and enhance asymmetry of the two subunits (Agniswamy et al., 2016; Cai et al., 1997; Cai et al., 2012; Cai et al., 2014; Galiano et al., 2009; Henes et al., 2019; Nakashima et al., 2016; Rajendran et al., 2023; Roche et al., 2015; Wong‐Sam et al., 2022; Zhang et al., 2014). These general conclusions are based on analyses of crystal structures, electron spin resonance data, NMR data, and molecular dynamics (MD) simulations, which have provided a consistent picture of wt‐PR dynamics. However, in studies of the inhibitor‐bound forms of the drug‐resistance mutants, NMR analysis of protein backbone amides point to minor conformational asymmetry between the two flaps in the mutants (Cai et al., 2014; Khan et al., 2018; Khan et al., 2021; Persons et al., 2018), which is in contrast to the significant structural and dynamical changes of the inhibitor‐bound PRs suggested by MD simulations (Agniswamy et al., 2016; Cai et al., 2014; Galiano et al., 2009; Henes et al., 2019; Nakashima et al., 2016; Rajendran et al., 2023; Wong‐Sam et al., 2022; Zhang et al., 2014).

The absence of significant conformational asymmetry in the flaps was deduced from small effects on backbone amide signals in the inhibitor‐bound PR and relates to the physics inherent to the specific spectroscopy approach. Amide ^1^H and ^15^N chemical shifts of diamagnetic proteins reflect electron densities in the local environment, at a level that these can be utilized for secondary‐structure prediction of proteins (Wishart et al., 1992). NMR relaxation, which has been used to study HIV‐1 PR dynamics, is sensitive to local rotational motion but not to translational motion (Dayie et al., 1996; Ishima & Torchia, 2000; Lipari & Szabo, 1981; Lipari & Szabo, 1982; Palmer III, 1997). In addition, NMR residual dipolar couplings (RDCs) of the protein backbone atoms are sensitive to relative changes of the orientation of the bond vector against the alignment tensor of the molecule (Roche et al., 2015). When these average properties dominate the data, it is difficult to detect small changes. Therefore, we hypothesized that NMR experiments using nuclei more sensitive to both chemical and local environments may help detect conformational changes in PRs upon drug‐resistance mutations, particularly in the inhibitor‐bound forms.

We tested this hypothesis using ^19^F NMR. ^19^F is extremely electronegative, rendering its chemical shifts very sensitive to small changes in the electronic environment. ^19^F NMR has been applied to characterize molecular interactions, conformational changes, and surface solvent effects in biomolecules (Emsley & Phillips, 1971; Gerig, 1994; Gronenborn, 2022; Kitevski‐LeBlanc & Prosser, 2012). We prepared wt‐PR and three drug‐resistance mutant proteins previously identified and characterized in a viral passage study against darunavir (DRV). The three mutant proteins contained either (i) four mutations (K45I, M46I, V82F, and I84V, Figure 1c); (ii) 10 mutations (those in [i] plus I13V, G16E, V32I, L33G, A71V, and L76V, Figure 1d) or (iii) 11 mutations (those in [ii] plus I54L, Table 1) (Henes et al., 2019). These proteins, referred to as 4Mut, 10Mut and 11Mut, respectively, along with wt protein were uniformly labeled with ^15^N and amino acid‐specifically labeled with ^19^F at the 5‐indole position (5F‐Trp). Two conserved Trp residues in PR reside at positions 6 and 42, ideally positioned to report on changes in the dimerization interface and the flap disposition (Figure 1c). Based on the crystal structure of PR (Henes et al., 2019), position 5 of W6 is near the dimer interface and solvent‐exposed. Position 5 of W42 is at the end of the flap β‐hairpin structure (residues from 43 to 58) and is partially buried in the protein toward the β‐hairpin (Figures 1c and S1, Supporting Information). Importantly, both Trp residues are at least 14 Å away from DRV and thus are expected to sense changes in the structure and/or dynamics of PR but not a direct effect from DRV.

**TABLE 1:** Mutations in the pathway to generate multi‐drug resistance.^a^

Variant	Mutations					
WT						
4Mut			K45I M46I			V82F I84V
10Mut	I13V G16E	V32I L33F	K45I M46I		A71V L76V	V82F I84V
11Mu	I13V G16E	V32I L33F	K45I M46I	I54L	A71V L76V	V82F I84V

^a^ Details are available in Henes et al. (2019).

Our 1D ^19^F NMR spectra of the PRs, both in the inhibitor‐free and DRV‐bound forms, showed that the W42 resonance in the mutants progressively shifted to a lower field, toward the cleaved‐product position. In addition, in the DRV‐bound form, the W42 resonance of 10Mut and 11Mut is split into two. ^19^F magic‐angle spinning (MAS) NMR spectra of DRV‐bound crystals of wt‐PR and 10Mut also exhibited three resonances, similar to the pattern in solution: one W6 resonance and two W42 resonances, with a lower‐field shift for one of the W42 resonances of 10Mut. Taken together, our results clearly demonstrate asymmetric flap‐inhibitor interaction in inhibitor‐bound forms, both in solution and in microcrystalline forms.

## 2 RESULTS

### 2.1 Folding of PRs and backbone dynamics in solution

5F‐Trp/U‐^15^N labeled PRs were expressed in BL21 (DE3) cells grown in modified M9 medium containing ^15^NH_4_Cl as the sole nitrogen source supplemented with 5‐fluoroindole before PR induction. Mass spectrometry analysis of the purified PRs revealed one major peak (85%–90% yield) and one minor peak (10%–15% yield). For each protein, the major fraction exhibited a molecular mass corresponding to 99% ^15^N labeling and 100% ^19^F labeling of the two Trp residues (Table S1). The minor fraction corresponded to 99% ^15^N labeling and ^19^F labeling for one of the two Trp positions (Figure S2). We concluded that this labeling efficiency was sufficient for our study.

**FIGURE 2:**
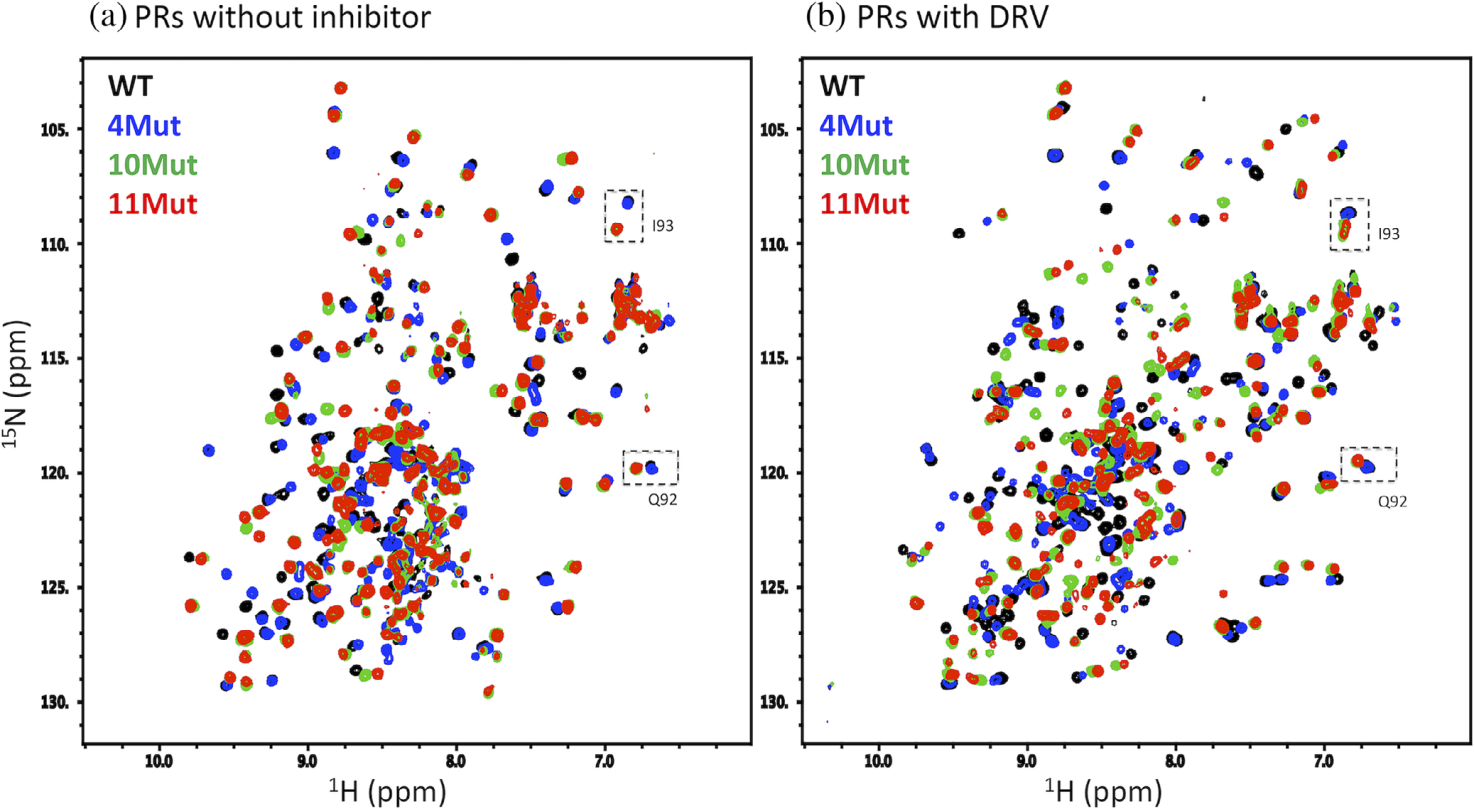
Superposition of ^1^H‐^15^N HSQC spectra of 5F‐Trp/U‐^15^N labeled wt‐PR, 4Mut, 10Mut, and 11Mut in (a) inhibitor‐free and (b) DRV‐bound forms. The protein concentrations were at 20–25 μM (as dimer) in 20 mM phosphate buffer at 293 K.

Proper folding of the PRs was confirmed by recording ^1^H‐^15^N HSQC spectra in solution (Figure 2a). All PR spectra were similar to those known from previous work on PR dimers, with ^1^H resonances for Q92 and I93 amides at 7 ppm or below, reflecting the known inter‐subunit ring‐current effect (Louis et al., 2003). Very weak indole NH resonances of the Trp residues were also observed (not shown in Figure 2a), consistent with the presence of a small fraction of protein having only one 5F‐Trp labeled (Figure S1). DRV binding altered a subset of the PR resonances in the ^1^H‐^15^N HSQC spectra, as observed previously (Figure 2b) (Khan et al., 2018; Persons et al., 2018).

**FIGURE 3:**
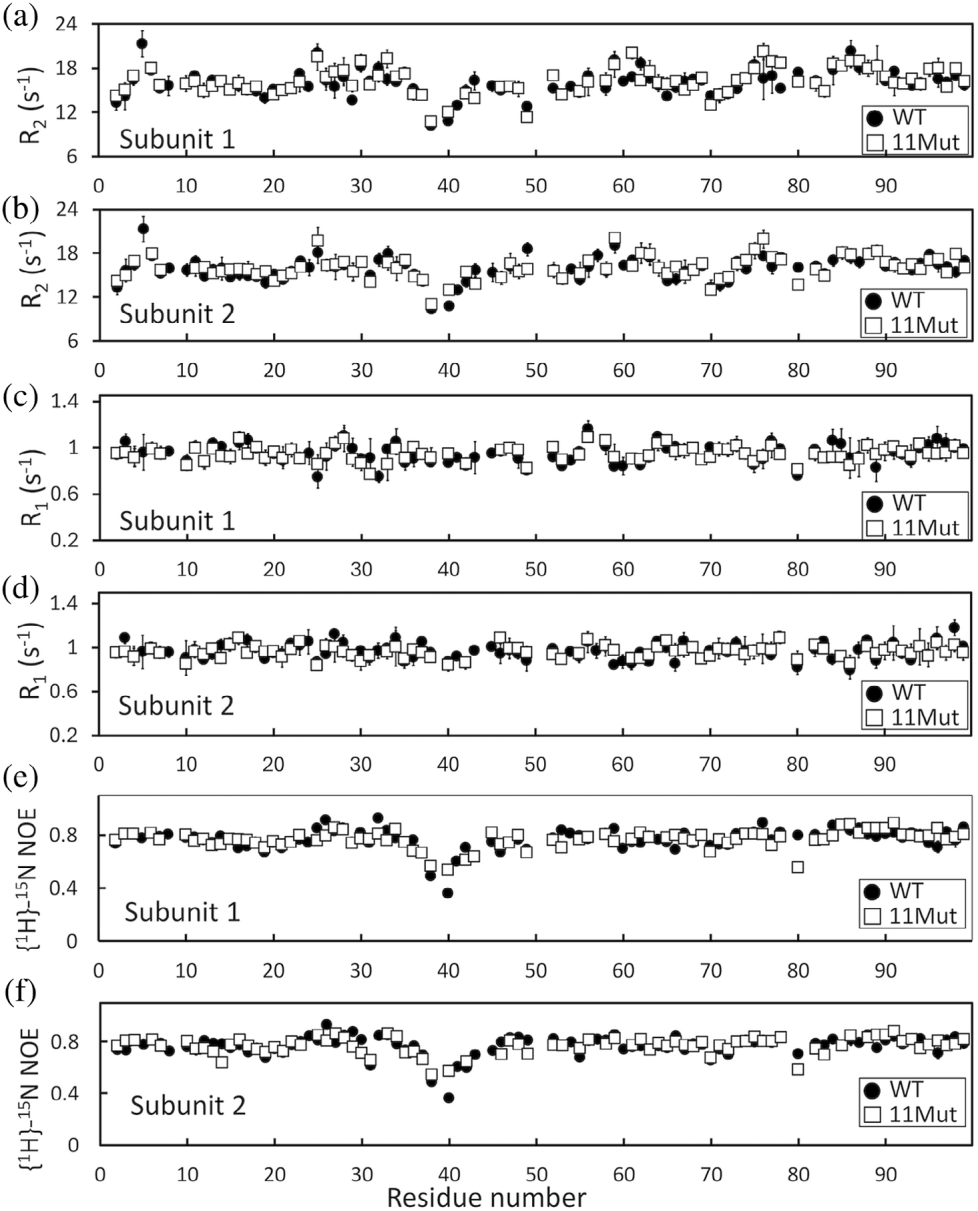
NMR relaxation data of 5F‐Trp/U‐^15^N labeled wt‐PR (black circles) and 11Mut (white rectangles) in DRV‐bound forms in solution: (a, b) transverse relaxation rate (R_2_), (c, d) longitudinal relaxation rate (R_1_), and (e, f) {^1^H}‐^15^N NOE values, each for subunit 1 (a, c, e) and subunit 2 (b, d, f). Note, some amino acids are tentatively assigned to either subunit 1 or 2 to enable comparison between wt‐PR and 11Mut (see Tables S2, S3). For residues with identical amide resonances in the two subunits, identical relaxation rates are plotted for both subunits.

Backbone dynamics of DRV‐bound wt‐PR and 11Mut were evaluated by recording ^15^N transverse relaxation rate (R_2_, Figure 3a,b), ^15^N longitudinal relaxation rate (R_1_, Figure 3c,d), and {^1^H}‐^15^N heteronuclear NOE (Figure 3e,f) data. To enable comparison of wt‐PR and 11Mut relaxation data, backbone resonance assignments were performed for DRV‐bound wt‐PR and 11Mut, and resonances were tentatively assigned to one of the two chains (for details, see footnotes of Tables S2, S3). The overall relaxation profile of wt‐PR was similar to that in a previous relaxation study, with only a few minor differences (Figure 3) (Cai et al., 1997). The presence of subtle differences is not surprising, since here the NL4‐3 wt‐PR (Henes et al., 2019) was used rather than the NMR‐optimized wt‐PR of the previous study (Cai et al., 2014) (see Materials and Methods).

Comparison of the wt‐PR relaxation profiles with those of 11Mut showed nearly identical data, with few exceptions (Figure 3). Residue 80 is positioned between two proline residues, forming a P79‐T80‐P81 stretch, and displayed slightly lower R_2_ and NOE in 11Mut compared to the wt‐PR, consistent with previous observations (Cai et al., 1997). In the current data, G40, located in a loop region called “elbow” (residues 38–42, Zoete et al., 2002), showed slightly lower R_2_ and NOE in the wt‐PR, compared to 11Mut (Figure 3). In the flap region (residues 43–58), no substantive differences in relaxation values between wt‐PR and 11Mut were noted (Figure 3). Overall, aside from a few sites, the NMR relaxation data generally reflect a rigid, inhibitor‐bound form of all PRs studied, even the PR containing the 11 mutations.

### 2.2 ^19^F NMR spectra reveal chemical shift changes for the PR mutants

**FIGURE 4:**
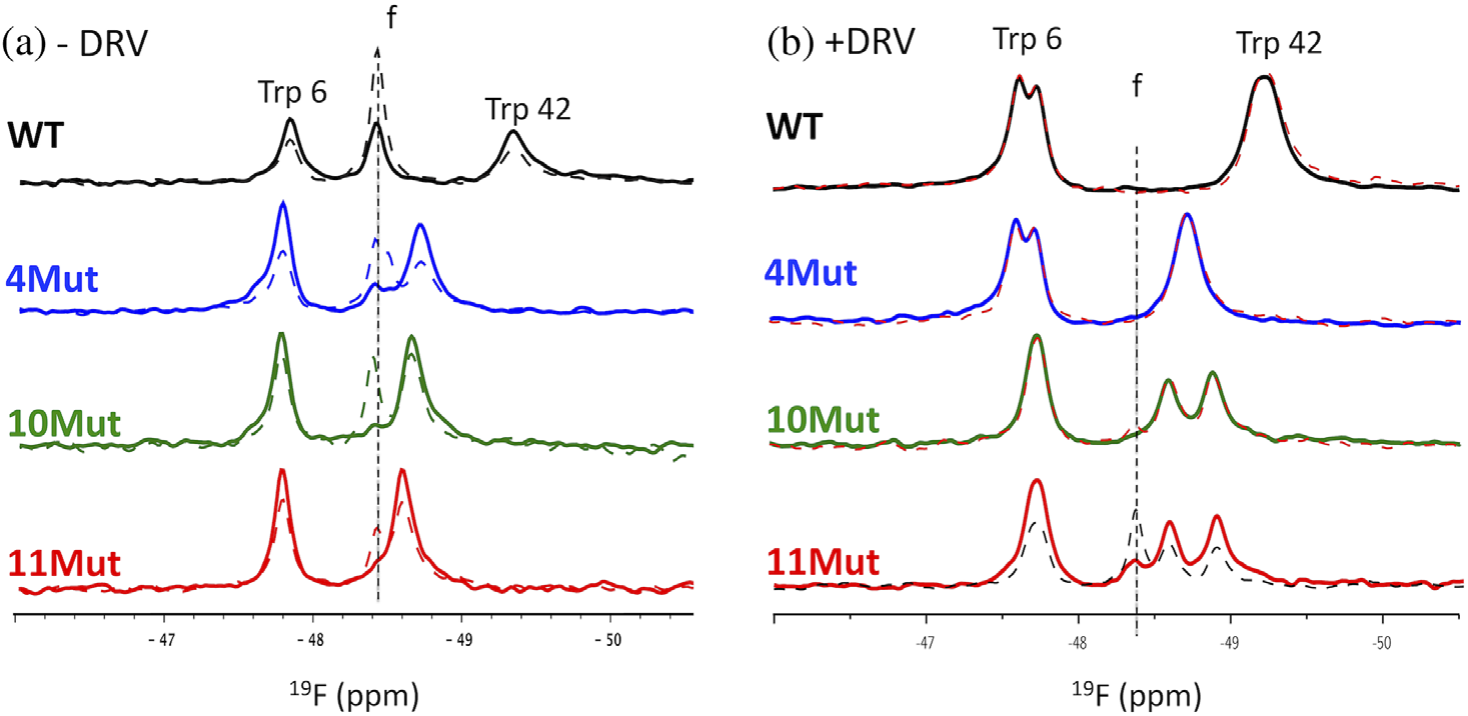
1D ^19^F NMR spectra of 5F‐Trp/U‐^15^N labeled wt‐PR (black), 4Mut (blue), 10Mut (green), and 11Mut (red) (a) without inhibitor and (b) with DRV in solution. In both panels, spectra of fresh samples are shown in solid lines, and those recorded 2–3 days later are shown in dashed lines. “f” indicates the resonance of proteolytic fragments.

^19^F NMR spectra were recorded for 5F‐Trp/U‐^15^N labeled PRs in their inhibitor‐free forms. The ^19^F NMR spectra of 4Mut, 10Mut, and 11Mut exhibited two resonances, one from W6 and the other from W42 (details below), while the spectrum of wt‐PR exhibited three resonances (Figure 4a). The middle resonance in the wt‐PR spectrum, at −48.4 ppm, increased in intensity over time, and similar signals were also observed over time for 4Mut, 10Mut, and 11Mut (Figure 4a, dashed lines), indicating that the middle resonance resulted from autoproteolysis of PRs. Considering that the k_cat_/K_m_ value of wt‐PR is approximately double that of 4Mut and more than 10 times higher than that of 11Mut (Henes et al., 2019), observation of autoproteolysis already in the fresh sample of wt‐PR was not surprising. The other two resonances, at −47.8 ppm and at −48.6 ~ −49.3 ppm showed similar peak intensities or areas across the various protein samples (Table S4), indicating that these two resonances were from W6 and W42.

Assignment of each 5F‐Trp resonance was based on the mutation sites and their locations relative to Trp resonances. For example, comparing the ^19^F NMR spectrum of wt‐PR with that of 4Mut, the resonance at −49.3 ppm in wt‐PR shifted downfield to −48.7 ppm in the 4Mut protein, while the resonance at −47.8 ppm remained essentially unchanged (Figure 4a and Table S4). The mutations in 4Mut are K45I, M46I, V82F, and I84V (Table 1, and Figure 1c), with residue 45 being the closest to W42 at 5.5 Å. Indeed, K45I and M46I are located within the same β‐strand as W42. In contrast, among the 4Mut mutations, the closest residue to W6 is residue 84 (17 Å). We therefore reasoned that the higher field resonance belongs to W42. Consistent with this, the ^19^F NMR spectra of 10Mut and 11Mut—the latter containing an I54L mutation on 10Mut—showed a slight difference in the chemical shift of the W42 resonance, at −48.62 ppm and −48.58 ppm, respectively, with no change in the W6 resonance position.

Importantly, the ^19^F chemical shift of the W42 resonance of PR changed with increasing numbers of amino acid changes, moving from −49.3 ppm to −48.6 ppm, toward the fragment peak position (Figure 4a and Table S4), while the chemical shift of the W6 resonance did not change much. Since PRs may weakly bind substrate/product generated by autoproteolysis, one might consider the possibility that the observed shift was due to the equilibrium of the bound/unbound forms. However, the chemical shift of the W42 resonance did not change by possible autoproteolysis (Figure 4a, dashed lines), making this possibility unlikely. The over 0.5 ppm change in the W42 resonance in 4Mut compared to wt‐PR may have been due to an electron‐shielding difference, since in the K45I mutation, a positive charge is removed.

### 2.3 ^19^F NMR reveals conformational asymmetry of the two flaps in the DRV‐bound form

^19^F NMR spectra recorded for 5F‐Trp/U‐^15^N labeled PRs in the presence of DRV exhibited patterns similar to those of the inhibitor‐free forms, that is, deshielding of the W42 resonance as the number of mutated residues increased (Figure 4b). In addition, the W42 resonance clearly split into two separate peaks in 10Mut and 11Mut (Figure 4b). The intensity ratio of W42 peaks was 0.25:0.28 and the area ratio was 0.23:0.32 (Table S5). Although the resonance intensity of the downfield W42 peak was slightly less than that of the upfield W42, the intensity ratio did not exhibit temperature‐dependent changes over a range of temperatures, from 283 K to 305 K (Figure S3 and Table S6). Therefore, the two W42 resonances stem from different environments of W42 in the two subunits, rather than two‐state dynamics of PR.

Since the chemical shift difference of the two W42 resonances in the DRV‐bound form was not observed in wt or 4Mut and observed only in 10Mut and 11Mut, the shift was not due to a direct effect of the inhibitor's electron distribution or induced magnetic field but instead arose from a structural difference in the PR subunits. This finding unambiguously revealed conformational asymmetry of the two flaps in the dimer. Unfortunately, the degree of conformational asymmetry cannot be determined from our data, considering the pin‐point observation of W42 and the sensitivity of ^19^F NMR to small environmental changes. At the very least, our observation of a conformational difference is qualitatively consistent with results from previous MD simulations, which suggest asymmetric flap structures in the inhibitor‐bound forms (Agniswamy et al., 2016; Cai et al., 2014; Galiano et al., 2009; Henes et al., 2019; Nakashima et al., 2016; Rajendran et al., 2023; Wong‐Sam et al., 2022; Zhang et al., 2014).

### 2.4 ^19^F solid‐state MAS NMR spectra of PR‐DRV crystals reveal a similar spectral pattern to that in solution

In order to investigate ^19^F NMR spectral pattern of 5F‐Trp in the crystalline state, we prepared crystals of 5F‐Trp/U‐^15^N labeled wt‐PR and 10Mut in their DRV‐bound forms and performed ^19^F MAS NMR experiments. 11Mut was not used in this crystallization study due to its instability (Figure 4b, dashed lines). Since the inhibitor‐free form of PR undergoes autoproteolysis, crystallization was only performed for the DRV‐bound and not the inhibitor‐free forms of the proteins.

**FIGURE 5:**
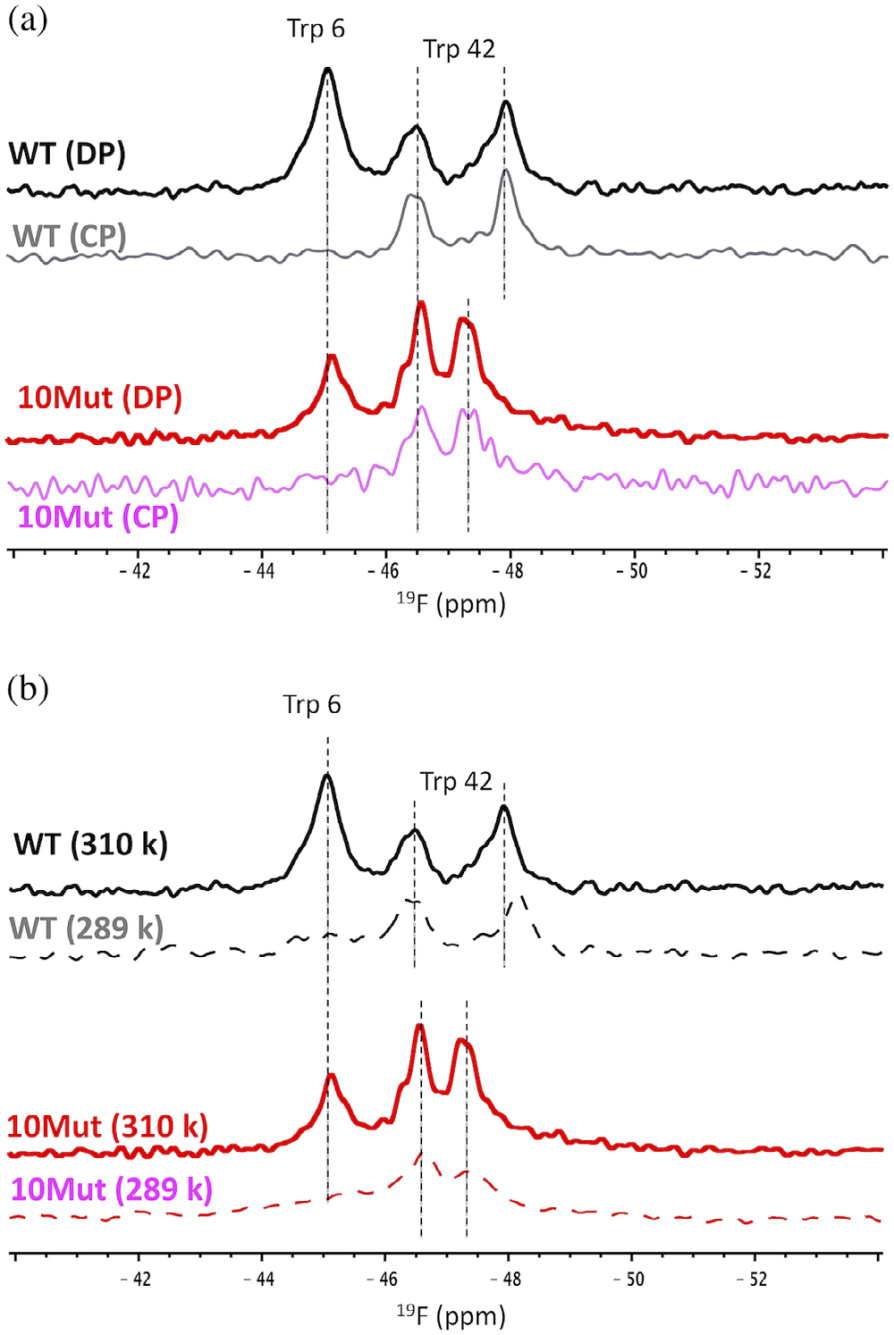
1D ^19^F MAS NMR spectra of 5F‐Trp/U‐^15^N labeled wt‐PR (black and gray) and 10Mut (red and pink) in crystalline DRV‐bound forms. (a) DPMAS (bold lines) and CPMAS (thin lines) spectra recorded at 310 K, and (b) DPMAS spectra recorded at 310 K (bold lines) and 289 K (dashed lines). In panel (b), spectra at 310 K (bold lines) are the same as those in (a).

Direct‐polarization MAS (DPMAS) spectra of the DRV‐bound wt‐PR and 10Mut crystals exhibited three resonances (Figure 5a). The resonance at −45 ppm was the same in both wt‐PR and 10Mut, suggesting that this resonance belongs to the W6 indole. The resonance at −48 ppm in the DRV‐bound wt‐PR moved in DRV‐bound 10Mut, similar to what was observed in solution, and was therefore assigned to W42. The ^1^H‐^19^F cross‐polarization (CPMAS) spectra showed a loss of intensity for the peak at −45 ppm (Figure 5a), but not for the other two resonances (described in more detail below). We concluded that the resonance at −45 ppm originated from the W6 indole, whereas the other two resonances were from W42.

The relative intensity of the W6 resonance, compared to that of the W42 resonance, in the ^19^F DPMAS spectrum of DRV‐bound wt‐PR differed from that of 10Mut (Figure 5a). Along with the decrease in intensity seen in the CPMAS data (Figure 5a), this indicates that the W6 region experiences internal motion. The dynamic nature of W6 was further confirmed by comparing ^19^F DPMAS spectra recorded at 310 K and 289 K. At 289 K, W6 intensity dropped below that of W42, and the peak nearly disappeared (Figure 5b). Similarly, DRV‐bound 10Mut showed a comparable spectral change at these two temperatures (Figure 5b). The observed dynamics of W6 align with the L5‐W6 peptide bond being the primary site of autoproteolysis (Rose et al., 1993) and with a previous NMR chemical exchange analysis of the L5‐W6 loop's dynamics in HIV‐1 PR (Ishima et al., 1999).

In addition, the ^19^F MAS NMR spectra of the crystalline 10Mut in the DRV‐bound form exhibited a resonance pattern similar to that in solution, and the W42 shift direction upon mutation was also similar, indicating different environments for the two W42 sites in 10Mut (Figure 4b). Significantly, the ^19^F MAS NMR spectra of DRV‐bound wt‐PR exhibited two resonances for W42, similar to the case of 10Mut in the crystalline state (Figure 5a), whereas the W42 resonance of DRV‐bound wt‐PR in solution did not split (Figure 4b). Interestingly, the −48 ppm resonance of crystalline wt‐PR in the DRV‐bound form at 310 K shifted upfield by 0.2 ppm at 289 K, a change not seen in the 10Mut (Figure 5b), indicating that the dynamic nature of the W42 site affects the chemical shift.

## 3 DISCUSSION

In this study, conformational dynamics of PR flaps in response to drug‐resistance mutations were investigated using ^19^F NMR spectroscopy, which is sensitive to small changes in chemical environment (Emsley & Phillips, 1971; Gerig, 1994; Gronenborn, 2022). W42 is located at the edge of the flap region, with the 5‐position of the indole ring facing the β‐hairpin structure. Thus, this fluorine is ideally suited to report the flap conformation (Figure S1c). In contrast, W6 is located near the dimer interface and exposed to the solution (Figure S1b). Our results from inhibitor‐free forms of these proteins revealed a progressive downfield shift of the W42 fluorine resonance as the number of mutation sites increased (Figure 4a), demonstrating a correlation between the fluorine chemical shift and the mutations along the drug‐resistance pathway. Given that a correlation between ^19^F chemical shift and folding is a standard technique in the protein folding field (Bann et al., 2002; Danielson & Falke, 1996; Ropson & Frieden, 1992), observation of such “directionality” in the ^19^F chemical shift changes as the PR drug‐resistance pathway evolves may suggest a small increase in flap dynamics or solvent exposure of the 5‐position of W42. This interpretation is consistent with previous observations that flap dynamics increase as drug‐resistance mutations accumulate (Roche et al., 2015; Zhang et al., 2014). In addition, the observation of similar shift changes in both the inhibitor‐free and bound forms (Figure 4) suggests that the ^19^F shift reflects electronic changes introduced by the mutations.

In the inhibitor‐free form, ^19^F chemical shifts of the W6 in 4Mut, 10Mut, and 11Mut changed only slightly, by ~0.1 ppm, compared to that of wt‐PR (Figure 4a and Table S4). 4Mut contains four mutations: K45I and M46I are located in the flap region, and V82F and I84V are located in a loop adjacent to the flap region (Figure 1c). Given that residues 45 and 46 are located in the flap region in PR, changes in the ensemble of flap conformations in 4Mut could affect the dimer interface that is reported on by the W6 resonance. Indeed, it is well known that flap regions interact and contribute to dimerization, and we previously showed that methyl chemical shifts for amino acids at the tips of the flaps differ between dimer and monomer (Ishima & Louis, 2008). Flap‐flap interaction in the PR dimer was also observed in NMR RDC experiments (Roche et al., 2015). Taken together with these previous observations, we conclude that the ^19^F chemical shifts of W6 sensitively detect a remote conformational change in the flap region at the dimer interface.

The mechanism by which a symmetric homodimer recognizes an asymmetric substrate or inhibitor has been a major focus in HIV‐1 PR structural biology (Dreyer et al., 1993; Prabu‐Jeyabalan et al., 2000, 2002). In the current study, we observed that the W42 resonance clearly split into two in the DRV‐bound forms of PR in solution for 10Mut and 11Mut (Figure 4b and Table S5). This observation is qualitatively consistent with results of previous MD simulations, which suggested asymmetric flap structures in the inhibitor‐bound forms (Agniswamy et al., 2016; Cai et al., 2014; Galiano et al., 2009; Henes et al., 2019; Nakashima et al., 2016; Rajendran et al., 2023; Wong‐Sam et al., 2022; Zhang et al., 2014). In particular, interdependence between subsites throughout HIV‐1 PR upon inhibitor binding has been repeatedly detected in simulations (Henes et al., 2019; Paulsen et al., 2017). Among the additional mutations in 10Mut compared to 4Mut, V32I and L76V directly interact with the flap region, and residue 32 directly interfaces with the inhibitor. Thus, we consider that the asymmetry in the W42 resonance is not present in 4Mut but is present in 10Mut and 11Mut.

Interestingly, an almost identical spectral pattern was observed in solution and in the crystalline state of DRV‐bound 10Mut. Although various experimental conditions differed between the solid and the solution state studies, the crystalline and solution ^19^F NMR spectra consistently showed two W42 resonances. Thus, it is reasonable to assume that the similarity of the spectral patterns stems from similar local structure characteristics, presumably flap asymmetry in the DRV‐bound form. A pair of W42 resonances was also observed in the crystalline state of wt‐PR, but not in solution. Given that dynamic averaging is more restricted in crystal forms than in solution, it comes as no surprise that the degree of conformational asymmetry was more pronounced in the crystal than in solution for the wt‐PR complex with DRV.

This study presents both ^15^N relaxation of the protein backbone and ^19^F chemical shifts at selected sites in HIV‐1 PRs. Our use of two NMR observables provided insight into the conformational characteristics of DRV‐bound WT and 11Mut (or 10Mut). Backbone ^15^N relaxation showed very similar dynamics throughout the protein between the two subunits, except for a few residues. Importantly, there was no significant reduction in the {^1^H}‐^15^N NOE in the flap region, indicating that the flap β‐hairpin structure did not melt, even in 11Mut (Figure 3). This observation is consistent with the flaps being predominantly closed in the DRV‐bound form in solution (Cai et al., 2014; Nicholson et al., 1995). Thus, the conformational asymmetry probed by ^19^F NMR cannot be due to significant differences in spatial motion. Previous studies using crystal structures and simulations also revealed very subtle changes in dynamic propagation through the protease upon mutation (Henes et al., 2019; Paulsen et al., 2017). Given that ^19^F NMR of proteins often provides only pinpoint information at the labeled sites, complementary protein NMR data are critical to avoid overinterpretation.

## 4 CONCLUSIONS

Structural asymmetry in HIV‐1 PR flaps for mutants along the drug resistance pathway was monitored using ^19^F NMR spectroscopy: the W42 resonance progressively shifted downfield with increasing numbers of amino acid changes, sensitively reporting on the asymmetric flap conformation in the inhibitor‐bound forms. The chemical shift difference was observed not only in solution but also, and more pronouncedly, in crystals of the fluorinated proteins in the presence of DRV. Overall, our data demonstrate that ^19^F NMR spectroscopy is a sensitive means of detecting subtle yet pivotal conformational differences in HIV‐1 PR associated with drug‐resistance mutations.

## 5 MATERIALS AND METHODS

### 5.1 Protein expression and purification

Genes encoding the following wt‐PR, and its three mutants, were prepared as described previously (Henes et al., 2019): PQITLWKRPL VTIKIGGQLK EALLDTGADD TVLEEMNLPG RWKPKMIGGI GGFIKVRQYD QILIEICGHK AIGTVLVGPT PVNIIGRNLL TQIGCTLNF. Compared to a previously used protein that was sequence‐optimized for NMR experimentation (Khan et al., 2018), the wt sequence used here was different, and the protein contained V32, R41, C67, and C95. 5F‐Trp/U‐^15^N labeled PRs were expressed in BL21 (DE3) cells transformed with the corresponding pET‐11a expression plasmids containing the relevant PR coding sequences and grown in modified M9‐defined medium with ^15^NH_4_Cl as a sole nitrogen source. Cells were grown at 37°C at 225 rpm up to OD 600 nm reached >0.9. The 5‐fluoroindole (CAS# 399‐52‐0, Millipore Sigma) was added 30 min before induction with 0.5 M β‐D‐1‐thiogalactopyranoside (IPTG). After 3–4 h incubation at 37°C, cells were harvested by centrifugation at 4000 rpm for 20 min at 4°C. The harvested cells were lysed by sonication, and PRs were purified from inclusion bodies, dissolved in 8 M urea, followed by fractionation in 8 M urea over gel filtration and HPLC using a previously established protocol (Ishima et al., 2007; Louis et al., 2009). Identity and purity of final proteins were assessed by SDS‐PAGE and mass spectrometry (a Bruker LC‐ESI‐TOF system, Bruker Daltonics Inc., Billerica, MA). Purified PRs were stored in the unfolded condition at −80°C. 5F‐Trp/U‐^15^N/U‐^13^C labeled wt‐PR and 11Mut were expressed and purified similarly, but by using modified M9 media containing ^13^C glucose.

PRs were folded before NMR experiments using the following protocol. Proteins were diluted with 20 mM formic acid at less than 0.3 mg/mL on ice, followed by further dilution with over threefold volume of 10 mM sodium acetate at pH 6.0 at room temperature. The PRs were dialyzed with 20 mM phosphate buffer at pH 5.8, containing 5 mM DTT. For the drug‐bound proteins, two‐fold excess DRV was added during folding, with an additional equimolar amount of DRV added before transferring the sample to the NMR tube.

### 5.2 Solution NMR experiments

All solution NMR experiments were performed at 293 K, unless indicated otherwise. 1D ^19^F NMR and 2D ^1^H‐^15^N HSQC spectra of 5F‐Trp/U‐^15^N labeled wt‐PR, 4Mut, 10Mut, and 11Mut were recorded on a Bruker 600 AVANCE spectrometer with a CP TXO F/C‐H‐D cryoprobe and a 5‐mm triple‐resonance cryoprobe, respectively. Proteins were prepared at 20–25 μM dimer protein concentration in the absence or presence of DRV, in 20 mM phosphate buffer at pH 5.8, containing 5 mM DTT. NMR experiments in the absence of inhibitor were performed in the following order: (1) 1D ^19^F NMR experiment, (2) 2D ^1^H‐^15^N HSQC experiments, (3) NMR 1D ^19^F NMR experiment. A set of two ^19^F NMR spectra was used to assess autoproteolytic cleavage of PRs. NMR experiments in the presence of DRV were performed in the following order: (1) 1D ^19^F experiment and (2) 2D ^1^H‐^15^N HSQC experiments. ^19^F NMR chemical shifts were referenced to trifluoracetic acid.

^15^N relaxation experiments for 5F‐Trp/U‐^15^N labeled wt‐PR and 11Mut were carried out at ~200 μM PR concentration in the presence of DRV on a Bruker 600 AVANCE spectrometer with a 5‐mm triple‐resonance cryoprobe. Resonance assignments of backbone amides of 5F‐Trp/U‐^15^N/U‐^13^C labeled wt‐PR and 11Mut were performed at ~200 μM PR concentration in the presence of DRV, using HNCACB experiments on a Bruker 800 AVANCE spectrometer, aided by comparison with available assignments in biological magnetic resonance bank (BMRB, 27727, 27731, 27377; see Tables S2, S3). Solution NMR data were processed and analyzed using NMRPipe (Delaglio et al., 1995), ccpNMR (Vranken et al., 2005), and exponential fits were carried out using Matlab software (The MathWorks Inc., Natick, MA).

### 5.3 Crystallization and MAS NMR experiments

Samples of 5F‐Trp/U‐^15^N labeled wt‐PR and 10Mut in the presence of DRV were prepared similarly to those for solution NMR, but with dialyzing PRs with 20 mM acetate buffer at pH 5.5, containing 5 mM DTT. Seed crystals were generated in a 24‐well plate (Hampton Research, Aliso Viejo, CA) using freshly prepared ~10 mg/mL PR complexed with DRV in 50 mM sodium citrate pH 5.2, 300 mM ammonium sulfate, 5 mM DTT. This condition was optimized from previous crystallization experiments (Adachi et al., 2009; Agniswamy et al., 2012; Nalam et al., 2013; Prabu‐Jeyabalan et al., 2002; Weber et al., 1997). The final microcrystalline samples were prepared by mixing 5 μL of the 10 mg/mL protein, 5 μL of the precipitant solution and 2.5 μL of the micro seeds mixture containing disruption glass beads at 0.5 mm size (Research Products International, Mount, Prospect, IL), and kept at 289 K. Microcrystals were packed into a 1.3 mm rotor for MAS NMR experiments.

^19^F MAS NMR experiments were performed with DRV‐bound crystals of wt‐PR and 10Mut on a 500 MHz Bruker AVIII spectrometer outfitted with a 1.3 mm HFX MAS probe. 1D ^19^F DPMAS and ^1^H‐^19^F CPMAS spectra were recorded at a MAS frequency of 60 kHz and T of 310 K (the actual sample temperature was calibrated using KBr as an external temperature sensor; Thurber & Tycko, 2009). 1D ^19^F MAS NMR spectra were also recorded at a MAS frequency of 40 kHz and T of 289 K. ^19^F chemical shifts were referenced using the high‐field mefloquine CF_3_ peak set at 8.8 ppm, used as a secondary reference (Guo et al., 2021).

## AUTHOR CONTRIBUTIONS

**Michel Guerrero:** Investigation; formal analysis; writing ‐ review and editing. **Roman Zadorozhnyi:** Investigation; formal analysis; writing – review and editing. **Guowu Lin:** Investigation; writing – review and editing. **Caitlin M. Quinn:** Investigation; writing – review and editing; methodology. **Guillermo Calero:** Investigation, wriging – review and editing; methodology; **Nese Kurt‐Yilmaz:** Conceptulation, resources, funding acquisition; writing ‐review and editing. **Celia A. Schiffer:** Conceptualization; resources; funding acquisition; supervision; writing – review and editing. **Tatyana Polenova:** Methodology; writing – review and editing; conceptualization; funding acquisition. **Angela M. Gronenborn:** Conceptualization; funding acquisition; supervision; methodology; writing – review and editing. **Rieko Ishima:** Conceptulation; investigation; supervision; writing – review and editing; writing – original draft.

## CONFLICT OF INTEREST STATEMENT

The authors declare no competing financial interests.

## Supporting information


**Table S1:** Mass numbers of 5F‐Trp/U‐^15^N labeled wt‐PR, 4Mut, 10Mut, and 11Mut.
**Table S2**: Backbone signal assignments of 5F‐Trp/U‐^15^N labeled wt‐PR in the DRV bound form, used to analyze the relaxation data in Figure 3.
**Table S3**: Backbone signal assignments of 5F‐Trp/U‐^15^N labeled 11Mut in the DRVbound form, used to analyze the relaxation data in Figure 3.
**Table S4**: ^19^F chemical shifts, intensities and areas of 5F‐Trp resonances of wt‐PR, 4Mut, 10Mut, and 11Mut in solution.
**Table S5**: ^19^F chemical shifts, intensities and areas of 5F‐Trp resonances of wt‐PR, 4Mut, 10Mut, and 11Mut in the DRV‐bound forms in solution.
**Table S6**: Temperature‐dependence of ^19^F chemical shifts and resonance intensities of 11Mut in the DRV‐bound form in solution.
**Figure S1**: Cartoon of (a) HIV‐1 wt‐PR showing positions of (b) W6 and (c) W42. In all panels, tryptophans are shown by sticks, and position 5 is marked in yellow. W6 indole is exposed to solution (panel b). W42 indole is partially buried in the protein core with R57 and Y59 on one side of the ring and P44 the other side.
**Figure S2**: Typical mass fractions of 5F‐Trp/U‐^15^N labeled wt‐PR, 4Mut, 10Mut and 11Mut, showing 10%–15% of the total having ^19^F‐labeling at only one Trp.
**Figure S3**: Temperature dependence of 11Mut in the presence of DRV in solution. “f” indicates fragment resonance. Note, chemical shift reference was adjusted at 293 K spectrum only.

## Data Availability

The data that supports the findings of this study are available in the supplementary material of this article.

## References

[pro70736-bib-0001] Adachi M , Ohhara T , Kurihara K , Tamada T , Honjo E , Okazaki N , et al. Structure of HIV‐1 protease in complex with potent inhibitor KNI‐272 determined by high‐resolution X‐ray and neutron crystallography. Proc Natl Acad Sci USA. 2009;106:4641–4646. 19273847 10.1073/pnas.0809400106 PMC2660780

[pro70736-bib-0002] Agniswamy J , Louis JM , Roche J , Harrison RW , Weber IT . Structural studies of a rationally selected multi‐drug resistant HIV‐1 protease reveal synergistic effect of distal mutations on flap dynamics. PLoS One. 2016;11:e0168616. 27992544 10.1371/journal.pone.0168616 PMC5161481

[pro70736-bib-0003] Agniswamy J , Shen CH , Aniana A , Sayer JM , Louis JM , Weber IT . HIV‐1 protease with 20 mutations exhibits extreme resistance to clinical inhibitors through coordinated structural rearrangements. Biochemistry. 2012;51:2819–2828. 22404139 10.1021/bi2018317 PMC3328860

[pro70736-bib-0004] Bann JG , Pinkner J , Hultgren SJ , Frieden C . Real‐time and equilibrium (19)F‐NMR studies reveal the role of domain‐domain interactions in the folding of the chaperone PapD. Proc Natl Acad Sci USA. 2002;99:709–714. 11792867 10.1073/pnas.022649599 PMC117370

[pro70736-bib-0005] Blackburn ME , Veloro AM , Fanucci GE . Monitoring inhibitor‐induced conformational population shifts in HIV‐1 protease by pulsed EPR spectroscopy. Biochemistry. 2009;48:8765–8767. 19691291 10.1021/bi901201q

[pro70736-bib-0006] Cai M , Zheng R , Caffrey M , Craigie R , Clore GM , Gronenborn AM . Solution structure of the N‐terminal zinc binding domain of HIV‐1 integrase. Nat Struct Biol. 1997;4:567–577. 9228950 10.1038/nsb0797-567

[pro70736-bib-0007] Cai Y , Myint W , Paulsen JL , Schiffer CA , Ishima R , Kurt Yilmaz N . Drug resistance mutations alter dynamics of inhibitor‐bound HIV‐1 protease. J Chem Theory Comput. 2014;10:3438–3448. 25136270 10.1021/ct4010454 PMC4132871

[pro70736-bib-0008] Cai Y , Yilmaz NK , Myint W , Ishima R , Schiffer CA . Differential flap dynamics in wild‐type and a drug resistant variant of HIV‐1 protease revealed by molecular dynamics and NMR relaxation. J Chem Theory Comput. 2012;8:3452–3462. 23144597 10.1021/ct300076y PMC3491577

[pro70736-bib-0009] Chetty S , Bhakat S , Martin AJ , Soliman ME . Multi‐drug resistance profile of PR20 HIV‐1 protease is attributed to distorted conformational and drug binding landscape: molecular dynamics insights. J Biomol Struct Dyn. 2016;34:135–151. 25671669 10.1080/07391102.2015.1018326

[pro70736-bib-0010] Coffin JM , Hughes SH , Varmus HE . The interactions of retroviruses and their hosts. In: Coffin JM , Hughes SH , Varmus HE , editors. Retroviruses. Cold Spring Harbor Laboratory Press: Cold Spring Harbor (NY), 1997. 21433350

[pro70736-bib-0011] Collins JR , Burt SK , Erickson JW . Flap opening in HIV‐1 protease simulated by ‘activated’ molecular dynamics. Nat Struct Biol. 1995;2:334–338. 7796268 10.1038/nsb0495-334

[pro70736-bib-0012] Danielson MA , Falke JJ . Use of 19F NMR to probe protein structure and conformational changes. Annu Rev Biophys Biomol Struct. 1996;25:163–195. 8800468 10.1146/annurev.bb.25.060196.001115 PMC2899692

[pro70736-bib-0013] Dayie KT , Wagner G , Lefevre JF . Theory and practice of nuclear spin relaxation in proteins. Annu Rev Phys Chem. 1996;47:243–282. 8930100 10.1146/annurev.physchem.47.1.243

[pro70736-bib-0014] Delaglio F , Grzesiek S , Vuister GW , Zhu G , Pfeifer J , Bax A . NMRPipe: a multidimensional spectral processing system based on UNIX pipes. J Biomol NMR. 1995;6:277–293. 8520220 10.1007/BF00197809

[pro70736-bib-0015] Deng NJ , Zheng W , Gallicchio E , Levy RM . Insights into the dynamics of HIV‐1 protease: a kinetic network model constructed from atomistic simulations. J Am Chem Soc. 2011;133:9387–9394. 21561098 10.1021/ja2008032 PMC3116037

[pro70736-bib-0016] Ding F , Layten M , Simmerling C . Solution structure of HIV‐1 protease flaps probed by comparison of molecular dynamics simulation ensembles and EPR experiments. J Am Chem Soc. 2008;130:7184–7185. 18479129 10.1021/ja800893d PMC3390170

[pro70736-bib-0017] Dreyer GB , Boehm JC , Chenera B , DesJarlais RL , Hassell AM , Meek TD , et al. A symmetric inhibitor binds HIV‐1 protease asymmetrically. Biochemistry. 1993;32:937–947. 8422397 10.1021/bi00054a027

[pro70736-bib-0018] Emsley JW , Phillips L . Fluorine chemical shifts. Prog Nucl Magn Reson Spectrosc. 1971;7:1–520.

[pro70736-bib-0019] Erickson JW , Burt SK . Structural mechanisms of HIV drug resistance. Annu Rev Pharmacol Toxicol. 1996;36:545–571. 8725401 10.1146/annurev.pa.36.040196.002553

[pro70736-bib-0020] Freedberg DI , Ishima R , Jacob J , Wang YX , Kustanovich I , Louis JM , et al. Rapid structural fluctuations of the free HIV protease flaps in solution: relationship to crystal structures and comparison with predictions of dynamics calculations. Protein Sci. 2002;11:221–232. 11790832 10.1110/ps.33202 PMC2373438

[pro70736-bib-0021] Galiano L , Ding F , Veloro AM , Blackburn ME , Simmerling C , Fanucci GE . Drug pressure selected mutations in HIV‐1 protease alter flap conformations. J Am Chem Soc. 2009;131:430–431. 19140783 10.1021/ja807531v PMC2705922

[pro70736-bib-0022] Gardner JM , Abrams CF . Energetics of flap opening in HIV‐1 protease: string method calculations. J Phys Chem B. 2019;123:9584–9591. 31640343 10.1021/acs.jpcb.9b08348 PMC7375464

[pro70736-bib-0023] Gerig JT . Fluorine NMR of proteins. Prog Nucl Magn Reson Spectrosc. 1994;26:293–370.

[pro70736-bib-0024] Ghosh AK , Chapsal BD , Weber IT , Mitsuya H . Design of HIV protease inhibitors targeting protein backbone: an effective strategy for combating drug resistance. Acc Chem Res. 2008;41:78–86. 17722874 10.1021/ar7001232

[pro70736-bib-0025] Gronenborn AM . Small, but powerful and attractive: (19)F in biomolecular NMR. Structure. 2022;30:6–14. 34995480 10.1016/j.str.2021.09.009 PMC8797020

[pro70736-bib-0026] Guo C , Fritz MP , Struppe J , Wegner S , Stringer J , Sergeyev IV , et al. Fast (19)F magic angle spinning NMR crystallography for structural characterization of fluorine‐containing pharmaceutical compounds. Anal Chem. 2021;93:8210–8218. 34080855 10.1021/acs.analchem.1c00784 PMC10280472

[pro70736-bib-0027] Harrison RW , Weber IT . Molecular dynamics simulations of HIV‐1 protease with peptide substrate. Protein Eng. 1994;7:1353–1363. 7700867 10.1093/protein/7.11.1353

[pro70736-bib-0028] Harte WE , Swaminathan S , Mansuri MM , Martin JC , Rosenberg IE , Beveridge DL . Domain communication in the dynamical structure of human immunodeficiency. Proc Natl Acad Sci USA. 1990;87:8864–8868. 2247458 10.1073/pnas.87.22.8864 PMC55060

[pro70736-bib-0029] Henes M , Lockbaum GJ , Kosovrasti K , Leidner F , Nachum GS , Nalivaika EA , et al. Picomolar to micromolar: elucidating the role of distal mutations in HIV‐1 protease in conferring drug resistance. ACS Chem Biol. 2019;14:2441–2452. 31361460 10.1021/acschembio.9b00370 PMC6941144

[pro70736-bib-0030] Hou T , Yu R . Molecular dynamics and free energy studies on the wild‐type and double mutant HIV‐1 protease complexed with amprenavir and two amprenavir‐related inhibitors: mechanism for binding and drug resistance. J Med Chem. 2007;50:1177–1188. 17300185 10.1021/jm0609162

[pro70736-bib-0031] Ishima R , Freedberg DI , Wang YX , Louis JM , Torchia DA . Flap opening and dimer‐interface flexibility in the free and inhibitor‐bound HIV protease, and their implications for function. Structure. 1999;7:1047–1055. 10508781 10.1016/s0969-2126(99)80172-5

[pro70736-bib-0032] Ishima R , Louis JM . A diverse view of protein dynamics from NMR studies of HIV‐1 protease flaps. Proteins. 2008;70:1408–1415. 17894346 10.1002/prot.21632

[pro70736-bib-0033] Ishima R , Torchia DA . Protein dynamics from NMR. Nat Struct Biol. 2000;7:740–743. 10966641 10.1038/78963

[pro70736-bib-0034] Ishima R , Torchia DA , Louis JM . Mutational and structural studies aimed at characterizing the monomer of HIV‐1 protease and its precursor. J Biol Chem. 2007;282:17190–17199. 17412697 10.1074/jbc.M701304200

[pro70736-bib-0035] Ishima R , Wingfield PT , Stahl SJ , Kaufman JD , Torchia DA . Using amide H‐1 and N‐15 transverse relaxation to detect millisecond time‐scale motions in perdeuterated proteins: application to HIV‐1 protease. J Am Chem Soc. 1998;120:10534–10542.

[pro70736-bib-0036] Kaplan AH , Zack JA , Knigge M , Paul DA , Kempf DJ , Norbeck DW , et al. Partial inhibition of the human immunodeficiency virus type 1 protease results in aberrant virus assembly and the formation of noninfectious particles. J Virol. 1993;67:4050–4055. 8510215 10.1128/jvi.67.7.4050-4055.1993 PMC237772

[pro70736-bib-0037] Karthik S , Senapati S . Dynamic flaps in HIV‐1 protease adopt unique ordering at different stages in the catalytic cycle. Proteins. 2011;79:1830–1840. 21465560 10.1002/prot.23008

[pro70736-bib-0038] Khan SN , Persons JD , Guerrero M , Ilina TV , Oda M , Ishima R . A synergy of activity, stability, and inhibitor‐interaction of HIV‐1 protease mutants evolved under drug‐pressure. Protein Sci. 2021;30:571–582. 33314454 10.1002/pro.4013 PMC7888579

[pro70736-bib-0039] Khan SN , Persons JD , Paulsen JL , Guerrero M , Schiffer CA , Kurt‐Yilmaz N , et al. Probing structural changes among analogous inhibitor‐bound forms of HIV‐1 protease and a drug‐resistant mutant in solution by nuclear magnetic resonance. Biochemistry. 2018;57:1652–1662. 29457713 10.1021/acs.biochem.7b01238 PMC5850901

[pro70736-bib-0040] Kitevski‐LeBlanc JL , Prosser RS . Current applications of 19F NMR to studies of protein structure and dynamics. Prog Nucl Magn Reson Spectrosc. 2012;62:1–33. 22364614 10.1016/j.pnmrs.2011.06.003

[pro70736-bib-0041] Kohl NE , Emini EA , Schleif WA , Davis LJ , Heimbach JC , Dixon RA , et al. Active human immunodeficiency virus protease is required for viral infectivity. Proc Natl Acad Sci USA. 1988;85:4686–4690. 3290901 10.1073/pnas.85.13.4686 PMC280500

[pro70736-bib-0042] Krausslich HG . Human immunodeficiency virus proteinase dimer as component of the viral polyprotein prevents particle assembly and viral infectivity. Proc Natl Acad Sci USA. 1991;88:3213–3217. 2014242 10.1073/pnas.88.8.3213 PMC51416

[pro70736-bib-0043] Lapatto R , Blundell T , Hemmings A , Overington J , Wilderspin A , Wood S , et al. X‐ray analysis of HIV‐1 proteinase at 2.7 A resolution confirms structural homology among retroviral enzymes. Nature. 1989;342:299–302. 2682266 10.1038/342299a0

[pro70736-bib-0044] Leidner F , Kurt Yilmaz N , Schiffer CA . Deciphering complex mechanisms of resistance and loss of potency through coupled molecular dynamics and machine learning. J Chem Theory Comput. 2021;17:2054–2064. 33783217 10.1021/acs.jctc.0c01244 PMC8164521

[pro70736-bib-0045] Lipari G , Szabo A . Nuclear magnetic resonance relaxation in nucleic acid fragments: models. Biochemistry. 1981;20:6250–6256. 7306511 10.1021/bi00524a053

[pro70736-bib-0046] Lipari G , Szabo A . Model‐free approach to the interpretation of nuclear magnetic resonance relaxation in macromolecules. 1. Theory and range of validity. J Am Chem Soc. 1982;104:4546–4559.

[pro70736-bib-0047] Louis JM , Ishima R , Aniana A , Sayer JM . Revealing the dimer dissociation and existence of a folded monomer of the mature HIV‐2 protease. Protein Sci. 2009;18:2442–2453. 19798742 10.1002/pro.261 PMC2821264

[pro70736-bib-0048] Louis JM , Ishima R , Nesheiwat I , Pannell LK , Lynch SM , Torchia DA , et al. Revisiting monomeric HIV‐1 protease. Characterization and redesign for improved properties. J Biol Chem. 2003;278:6085–6092. 12468541 10.1074/jbc.M209726200

[pro70736-bib-0049] Louis JM , Ishima R , Torchia DA , Weber IT . HIV‐1 protease: structure, dynamics, and inhibition. Adv Pharmacol. 2007;55:261–298. 17586318 10.1016/S1054-3589(07)55008-8

[pro70736-bib-0050] Nakashima M , Ode H , Suzuki K , Fujino M , Maejima M , Kimura Y , et al. Unique flap conformation in an HIV‐1 protease with high‐level darunavir resistance. Front Microbiol. 2016;7:61. 26870021 10.3389/fmicb.2016.00061 PMC4737996

[pro70736-bib-0051] Nalam MN , Ali A , Reddy GS , Cao H , Anjum SG , Altman MD , et al. Substrate envelope‐designed potent HIV‐1 protease inhibitors to avoid drug resistance. Chem Biol. 2013;20:1116–1124. 24012370 10.1016/j.chembiol.2013.07.014 PMC3934494

[pro70736-bib-0052] Navia MA , Fitzgerald PM , McKeever BM , Leu CT , Heimbach JC , Herber WK , et al. Three‐dimensional structure of aspartyl protease from human immunodeficiency virus HIV‐1. Nature. 1989;337:615–620. 2645523 10.1038/337615a0

[pro70736-bib-0053] Nicholson LK , Yamazaki T , Torchia DA , Grzesiek S , Bax A , Stahl SJ , et al. Flexibility and function in HIV‐1 protease. Nat Struct Biol. 1995;2:274–280. 7796263 10.1038/nsb0495-274

[pro70736-bib-0054] Oroszlan S , Luftig RB . Retroviral proteinases. Curr Top Microbiol Immunol. 1990;157:153–185. 2203608 10.1007/978-3-642-75218-6_6

[pro70736-bib-0055] Palmer AG III . Probing molecular motion by NMR. Curr Opin Struct Biol. 1997;7:732–737. 9345634 10.1016/s0959-440x(97)80085-1

[pro70736-bib-0056] Paulsen JL , Leidner F , Ragland DA , Kurt Yilmaz N , Schiffer CA . Interdependence of inhibitor recognition in HIV‐1 protease. J Chem Theory Comput. 2017;13:2300–2309. 28358514 10.1021/acs.jctc.6b01262 PMC5425943

[pro70736-bib-0057] Perryman AL , Lin JH , McCammon JA . Restrained molecular dynamics simulations of HIV‐1 protease: the first step in validating a new target for drug design. Biopolymers. 2006;82:272–284. 16508951 10.1002/bip.20497

[pro70736-bib-0058] Persons JD , Khan SN , Ishima R . An NMR strategy to detect conformational differences in a protein complexed with highly analogous inhibitors in solution. Methods. 2018;148:9–18. 29656080 10.1016/j.ymeth.2018.04.005 PMC6133750

[pro70736-bib-0059] Prabu‐Jeyabalan M , Nalivaika E , Schiffer CA . How does a symmetric dimer recognize an asymmetric substrate? A substrate complex of HIV‐1 protease. J Mol Biol. 2000;301:1207–1220. 10966816 10.1006/jmbi.2000.4018

[pro70736-bib-0060] Prabu‐Jeyabalan M , Nalivaika E , Schiffer CA . Substrate shape determines specificity of recognition for HIV‐1 protease: analysis of crystal structures of six substrate complexes. Structure. 2002;10:369–381. 12005435 10.1016/s0969-2126(02)00720-7

[pro70736-bib-0061] Quinones‐Mateu ME , Moore‐Dudley DM , Jegede O , Weber J , Eric JA . Viral drug resistance and fitness. Adv Pharmacol. 2008;56:257–296. 18086415 10.1016/S1054-3589(07)56009-6

[pro70736-bib-0062] Ragland DA , Whitfield TW , Lee SK , Swanstrom R , Zeldovich KB , Kurt‐Yilmaz N , et al. Elucidating the interdependence of drug resistance from combinations of mutations. J Chem Theory Comput. 2017;13:5671–5682. 28915040 10.1021/acs.jctc.7b00601 PMC5927819

[pro70736-bib-0063] Rajendran M , Ferran MC , Mouli L , Babbitt GA , Lynch ML . Evolution of drug resistance drives destabilization of flap region dynamics in HIV‐1 protease. Biophys Rep. 2023;3:100121. 10.1016/j.bpr.2023.100121 PMC10469570 37662576

[pro70736-bib-0064] Roche J , Louis JM , Bax A . Conformation of inhibitor‐free HIV‐1 protease derived from NMR spectroscopy in a weakly oriented solution. Chembiochem. 2015;16:214–218. 25470009 10.1002/cbic.201402585 PMC4293325

[pro70736-bib-0065] Ropson IJ , Frieden C . Dynamic NMR spectral analysis and protein folding: identification of a highly populated folding intermediate of rat intestinal fatty acid‐binding protein by 19F NMR. Proc Natl Acad Sci USA. 1992;89:7222–7226. 1496015 10.1073/pnas.89.15.7222 PMC49678

[pro70736-bib-0066] Rose JR , Salto R , Craik CS . Regulation of autoproteolysis of the HIV‐1 and HIV‐2 proteases with engineered amino acid substitutions. J Biol Chem. 1993;268:11939–11945. 8505318

[pro70736-bib-0067] Seelmeier S , Schmidt H , Turk V , von der Helm K . Human immunodeficiency virus has an aspartic‐type protease that can be inhibited by pepstatin A. Proc Natl Acad Sci USA. 1988;85:6612–6616. 3045820 10.1073/pnas.85.18.6612 PMC282027

[pro70736-bib-0068] Shen CH , Chang YC , Agniswamy J , Harrison RW , Weber IT . Conformational variation of an extreme drug resistant mutant of HIV protease. J Mol Graph Model. 2015;62:87–96. 26397743 10.1016/j.jmgm.2015.09.006 PMC4695280

[pro70736-bib-0069] Silva AM , Cachau RE , Sham HL , Erickson JW . Inhibition and catalytic mechanism of HIV‐1 aspartic protease. J Mol Biol. 1996;255:321–346. 8551523 10.1006/jmbi.1996.0026

[pro70736-bib-0070] Spinelli S , Liu QZ , Alzari PM , Hirel PH , Poljak RJ . The three‐dimensional structure of the aspartyl protease from the HIV‐1 isolate BRU. Biochimie. 1991;73:1391–1396. 1799632 10.1016/0300-9084(91)90169-2

[pro70736-bib-0071] Surleraux DL , de Kock HA , Verschueren WG , Pille GM , Maes LJ , Peeters A , et al. Design of HIV‐1 protease inhibitors active on multidrug‐resistant virus. J Med Chem. 2005;48:1965–1973. 15771440 10.1021/jm049454n

[pro70736-bib-0072] Thurber KR , Tycko R . Measurement of sample temperatures under magic‐angle spinning from the chemical shift and spin–lattice relaxation rate of 79Br in KBr powder. J Magn Reson. 2009;196:84–87. 18930418 10.1016/j.jmr.2008.09.019 PMC2632797

[pro70736-bib-0073] Tiefenbrunn T , Forli S , Baksh MM , Chang MW , Happer M , Lin YC , et al. Small molecule regulation of protein conformation by binding in the flap of HIV protease. ACS Chem Biol. 2013;8:1223–1231. 23540839 10.1021/cb300611p PMC3769432

[pro70736-bib-0074] Toth G , Borics A . Flap opening mechanism of HIV‐1 protease. J Mol Graph Model. 2006;24:465–474. 16188477 10.1016/j.jmgm.2005.08.008

[pro70736-bib-0075] Tzoupis H , Leonis G , Avramopoulos A , Mavromoustakos T , Papadopoulos MG . Systematic molecular dynamics, MM‐PBSA, and ab initio approaches to the saquinavir resistance mechanism in HIV‐1 PR due to 11 double and multiple mutations. J Phys Chem B. 2014;118:9538–9552. 25036111 10.1021/jp502687q

[pro70736-bib-0076] Ung PM , Dunbar JB Jr , Gestwicki JE , Carlson HA . An allosteric modulator of HIV‐1 protease shows equipotent inhibition of wild‐type and drug‐resistant proteases. J Med Chem. 2014;57:6468–6478. 25062388 10.1021/jm5008352 PMC4136727

[pro70736-bib-0077] Venkatachalam S , Muralidharan N , Pandian R , Sayed Y , Gromiha MM . Integrative computational approaches for understanding drug resistance in HIV‐1 protease subtype C. Viruses. 2025;17:850. 40573441 10.3390/v17060850 PMC12197794

[pro70736-bib-0078] Vranken WF , Boucher W , Stevens TJ , Fogh RH , Pajon A , Llinas M , et al. The CCPN data model for NMR spectroscopy: development of a software pipeline. Proteins. 2005;59:687–696. 15815974 10.1002/prot.20449

[pro70736-bib-0079] Wang R , Zheng Q . Multiple molecular dynamics simulations and free‐energy predictions uncover the susceptibility of variants of HIV‐1 protease against inhibitors darunavir and KNI‐1657. Langmuir. 2021;37:14,407–14,418. 10.1021/acs.langmuir.1c02348 34851643

[pro70736-bib-0080] Weber IT , Wu J , Adomat J , Harrison RW , Kimmel AR , Wondrak EM , et al. Crystallographic analysis of human immunodeficiency virus 1 protease with an analog of the conserved CA‐p2 substrate—interactions with frequently occurring glutamic acid residue at P2′ position of substrates. Eur J Biochem. 1997;249:523–530. 9370363 10.1111/j.1432-1033.1997.00523.x

[pro70736-bib-0081] Wishart DS , Sykes BD , Richards FM . The chemical shift index: a fast and simple method for the assignment of protein secondary structure through NMR spectroscopy. Biochemistry. 1992;31:1647–1651. 1737021 10.1021/bi00121a010

[pro70736-bib-0082] Wlodawer A , Erickson JW . Structure‐based inhibitors of HIV‐1 protease. Annu Rev Biochem. 1993;62:543–585. 8352596 10.1146/annurev.bi.62.070193.002551

[pro70736-bib-0083] Wlodawer A , Miller M , Jaskolski M , Sathyanarayana BK , Baldwin E , Weber IT , et al. Conserved folding in retroviral proteases: crystal structure of a synthetic HIV‐1 protease. Science. 1989;245:616–621. 2548279 10.1126/science.2548279

[pro70736-bib-0084] Wong‐Sam A , Wang YF , Kneller DW , Kovalevsky AY , Ghosh AK , Harrison RW , et al. HIV‐1 protease with 10 lopinavir and darunavir resistance mutations exhibits altered inhibition, structural rearrangements and extreme dynamics. J Mol Graph Model. 2022;117:108315. 36108568 10.1016/j.jmgm.2022.108315 PMC10091457

[pro70736-bib-0085] York DM , Darden TA , Pedersen LG , Anderson MW . Molecular dynamics simulation of HIV‐1 protease in a crystalline. Biochemistry. 1993;32:1443–1453. 8431424 10.1021/bi00057a007

[pro70736-bib-0086] Zhang Y , Chang YC , Louis JM , Wang YF , Harrison RW , Weber IT . Structures of darunavir‐resistant HIV‐1 protease mutant reveal atypical binding of darunavir to wide open flaps. ACS Chem Biol. 2014;9:1351–1358. 24738918 10.1021/cb4008875 PMC4076034

[pro70736-bib-0087] Zoete V , Michielin O , Karplus M . Relation between sequence and structure of HIV‐1 protease inhibitor complexes: a model system for the analysis of protein flexibility. J Mol Biol. 2002;315:21–52. 11771964 10.1006/jmbi.2001.5173

