# Supplementary material for: Conformational asymmetry of human immunodeficiency virus‐1 protease flaps increases along the darunavir drug resistance pathway
Source: Protein Sci. 2026 Aug 5;35(9):e70736. doi: 10.1002/pro.70736 (PMC13439748; doi:10.1002/pro.70736)
Supplement: Supplementary file 1 [file PRO-35-e70736-s001.pdf]

Conformational asymmetry of HIV-1 Protease flaps increases along the DRV drug resistance pathway

Michel Guerrero[1], Roman Zadorozhnyi[2,3], Guowu Lin[1], Caitlin M. Quinn[2], Guillermo Calero[1], Nese Kurt-Yilmaz[4], Celia A. Schiffer[4], Tatyana Polenova[2,3], Angela M Gronenborn[1,3], Rieko Ishima[1]*

[1]Department of Structural Biology, University of Pittsburgh School of Medicine, Pittsburgh, PA, USA

[2]Department of Chemistry and Biochemistry, University of Delaware, Newark, Delaware, USA.

[3]Pittsburgh Center for HIV Protein Interactions, University of Pittsburgh School of Medicine

[4]Department of Biochemistry and Molecular Biotechnology, University of Massachusetts Chan Medical School, Worcester, MA, USA

[*]Corresponding Author: Rieko Ishima, Room 1037, Biomedical Science Tower 3, 3501 Fifth Avenue, Pittsburgh, PA 15260. Tel: 412-648-9056. Email: ishima@pitt.edu

Table S1. Mass numbers of 5F-Trp/U-^{15}N labeled wt-PR, 4Mut, 10Mut and 11Mut.

**PR**	**Expected mass[1]**	**Measured mass**
5F-Trp/U-^{15}N		
Wt-PR	10983.55	10982.9
4Mut	10983.53	10982.8
10Mut	11103.64	11102.9
11Mut	11103.64	11102.9
5F-Trp/U-^{15}N/U-^{13}C		
Wt-PR	11449.05	11453.6
11Mut	11579.92	11580.5

1. Expected mass for 5F-Trp/U-^{15}N empirically assumes 99% ^{15}N labeling, and that for 5F-Trp/U-^{15}N/U-^{13}C assumes 99% ^{15}N labeling and 98% ^{13}C labeling.

Table S2. Backbone signal assignments of 5F-Trp/U-^{15}N labeled wt-PR in the DRV-bound form, used to analyze the relaxation data in **Figure 3**.

		Chain A (ppm)				Chain B (ppm)				Not splitting (ppm)			
No.	AA	H	N	CA	CB	H	N	CA	CB	H	N	CA	CB
1	P											62.10	31.24
2	Q									8.51	120.27	55.55	
3	I									9.51	129.06	60.76	40.06
4	T									8.42	116.16	60.94	70.56
5	L									9.09	116.36	54.14	40.93
6	W									7.33	120.84	58.84	28.88
7	K									7.47	115.10	53.56	35.40

		Chain 1 (ppm)				Chain 2 (ppm)				Not splitting (ppm)			
No.	AA	H	N	CA	CB	H	N	CA	CB	H	N	CA	CB
8	R	8.82	121.10	56.23		8.77	120.41	56.21	30.08				
9	P			61.64				61.62	28.84				
10	L	7.63	127.08	53.62	43.57	7.69	126.54	53.70	43.79				
11	V	9.24	116.34	58.72	35.92	9.20	116.13	58.72	36.01				
12	T	8.43	117.06	62.78	69.55	8.39	117.02	62.96	69.53				
13	I	9.29	121.38	58.37	40.91	9.27	120.80	58.37	40.96				
14	K	8.47	121.09	54.67	35.33	8.55	120.62	54.74	35.67				
15	I	8.93	125.06	59.08	40.39	8.91	124.98	58.83	40.59				
16	G	9.69	118.89	46.99		9.66	119.37	47.02					
17	G									8.83	106.11	44.99	
18	Q									7.97	119.70	54.48	31.54
19	L									8.46	123.20	54.34	42.75
20	K									8.90	122.33	53.92	37.26

		Chain A (ppm)				Chain B (ppm)				Not splitting (ppm)			
No.	AA	H	N	CA	CB	H	N	CA	CB	H	N	CA	CB
21	E	8.64	121.25	55.21	32.14	8.64	120.49	55.06	32.16				
22	A	9.37	125.88	50.62	24.50	9.40	125.92	50.77	25.08				
23	L	8.78	122.10	53.27	45.90	8.72	121.04	53.77	46.15				
24	L	8.40	125.34	55.88	40.21	8.25	122.72	54.88	40.61				
25	D	8.68	128.86	52.06	40.53	8.64	126.93	53.02	43.60				
26	T	8.17	111.08	65.73	68.73	8.38	112.73	64.73	69.02				
27	G	7.84	108.95	45.36		9.47	109.51	45.01					
28	A	7.62	126.64	50.14	20.08	6.92	124.56	49.15	22.05				
29	D	8.74	125.96	57.62	40.86	8.50	124.52	58.48	41.50				
30	D	7.60	115.82	53.10	44.95	7.19	112.89	52.54	45.87				
31	T	8.24	117.11	63.21	70.89	7.69	119.53	64.13	71.93				
32	V	8.31	127.83	60.14	33.70	9.05	127.36	59.06	34.47				
33	L	9.36	126.15	51.95	45.07	9.30	126.54	51.98	44.88				

34	E	8.03	115.42	55.42	30.30	8.17	115.76	55.18	30.67				
35	E	7.42	116.34	58.42	30.07	7.67	115.85	58.37	29.89				
36	M	7.00	120.24	54.68	34.02	7.01	120.18	54.56	33.95				
37	N	8.63	118.58	53.64	38.47	8.67	118.85	52.58	38.35				
38	L	7.30	124.59	51.95	42.83	7.39	124.67	51.85	43.05				

		Chain 1 (ppm)				Chain 2 (ppm)				Not splitting (ppm)			
No.	AA	H	N	CA	CB	H	N	CA	CB	H	N	CA	CB
39	P												
40	G									8.39	106.20	44.36	
41	R									8.42	118.86	56.11	31.10
42	W	7.48	117.50	53.77	31.05	7.50	118.08	53.87	31.05				
43	K	8.22	117.87	53.02	34.93	8.32	117.98	53.03	34.88				
44	P												

		**Chain A (ppm)**				**Chain B (ppm)**				Not splitting (ppm)			
No.	AA	H	N	CA	CB	H	N	CA	CB	H	N	CA	CB
45	K	8.53	121.75	55.64	36.74	8.60	121.49	55.50	36.84				
46	M	8.38	122.06	53.73	33.94	8.38	121.22	54.28	34.76				
47	I	8.89	118.30	58.60	42.58	8.65	116.35	59.89	41.76				
48	G	8.29	111.73	44.09		8.48	108.43	44.67					
49	G	7.48	106.82	43.73		7.45	107.00	45.24					
50	I												
51	G												
52	G	6.91	105.94	44.12		7.26	104.94	44.37					
53	F	8.63	118.48	57.17	42.19	8.45	118.62	56.12	42.87				
54	I	9.02	113.02	59.69	42.41	9.04	112.63	59.70	42.41				
55	K	8.41	122.92	56.07	33.28	8.46	122.97	56.16					
56	V	9.01	116.09	58.13	35.66	8.92	115.77	58.09	35.68				
57	R	8.87	118.43	55.57	32.22	9.05	118.80	55.34	32.17				
58	Q	9.78	123.68	55.70	29.96	9.85	123.28	55.97	30.25				

		Chain 1 (ppm)				Chain 2 (ppm)				Not splitting (ppm)			
No.	AA	H	N	CA	CB	H	N	CA	CB	H	N	CA	CB
59	Y									9.19	128.90	57.42	40.64
60	D	8.97	120.55	53.77	43.69	8.91	121.01	53.76	43.54				
61	Q	8.84	114.36	56.40	26.88	8.96	114.32	56.50	26.57				
62	I	8.63	122.18	57.42	36.63	8.69	121.76	57.55	36.93				
63	L	8.00	127.23	54.76	42.77	8.03	127.13	54.89	42.39				
64	I	9.21	126.54	59.89	41.84	9.27	127.10	59.47	42.05				
65	E									8.36	124.38	54.58	31.89
66	I	9.03	124.71	60.50	40.28	9.11	125.39	60.63	40.05				
67	C	9.13	126.11	59.84	28.50	9.27	126.17	59.85	28.46				

68	G	8.83	104.29	45.40		8.77	104.01	45.31					
69	H									8.17	119.03	54.46	29.63
70	K	8.97	125.00	57.47	33.29	8.93	124.68	57.28	33.40				
71	A	8.85	127.27	51.03	22.78	8.89	126.78	50.83	22.65				
72	I	8.44	119.50	58.94	40.93	8.40	119.44	58.86	40.64				
73	G	8.80	113.30	45.98		8.82	112.91	45.95					
74	T	8.60	118.53	63.88	70.11	8.70	118.38	63.32	70.43				
75	V	8.97	124.41	61.62	36.39	9.24	125.54	61.29	35.83				
76	L	8.23	125.49	52.31	41.26	8.20	124.75	52.29	41.46				
77	V	9.21	120.08	60.03	35.78	9.13	120.71	59.89	35.32				
78	G	8.97	113.20	45.97		9.24	114.52	46.06					
79	P												
80	T	8.34	118.99	57.03	69.80	8.40	120.07	56.80	70.29				
81	P												

		Chain A (ppm)				Chain B (ppm)				Not splitting (ppm)			
No.	AA	H	N	CA	CB	H	N	CA	CB	H	N	CA	CB
82	V	7.18	115.94	60.02	36.26	6.98	113.71	59.39	35.70				
83	N	8.59	125.01	53.75	38.36	8.48	122.37	54.24	38.48				
84	I	9.41	121.07	60.00	41.39	9.56	126.68	61.92	42.44				
85	I	8.80	123.28		35.71	9.27	126.19						
86	G	8.11	113.43	43.47		8.02	112.18	43.38					
87	R	9.35	116.58	61.28	30.00	9.18	116.68	61.35	29.72				
88	N	7.96	115.77	57.02	36.68	7.89	115.02	56.84	37.39				
89	L	6.96	116.23	55.45	42.68	7.01	115.64	55.40	42.20				

		Chain 1 (ppm)				Chain 2 (ppm)				Not splitting (ppm)			
No.	AA	H	N	CA	CB	H	N	CA	CB	H	N	CA	CB
90	L									8.20	119.25	57.84	41.76
91	T	7.92	106.42	63.67	68.05	7.88	106.11	63.64	68.21				
92	Q									6.72	119.67	57.49	19.10
93	I									6.85	108.60	60.35	37.54
94	G									7.17	107.50	46.06	
95	C	7.34	117.14	58.76	28.16	7.40	117.83	58.46	28.04				
96	T									9.20	117.50	59.79	72.40
97	L	8.49	119.56	53.47	45.46	8.44	120.12	53.44	45.66				
98	N	8.85	120.94	53.21	42.20	8.75	119.83	52.98	42.18				
99	F	8.00	122.18			7.99	121.79	58.82	38.54				

Footnote: The three types of residues are classified as follows: residues whose amide ^{15}N and ^{1}H chemical shifts (1) do not split upon binding to DRV, (2) split but subunits A and B are indistinguishable in PR dimer, and the residue is therefore listed under Chain 1 or Chain 2 in the Table, or (3) split and subunits A and B are distinguishable based on inhibitor-PR NOEs determined previously for wt-PR. In the latter case, the residues are listed under Chain A or Chain B, with Chain A in close proximity to a bis-tetrahydrofuranyl (bis-THF) carbamate moiety of DRV, consistent with previous reports (Henes et al., 2019; Khan et al., 2021; Khan et al., 2018; Paulsen et al., 2017; Persons et al., 2018)).

Table S3. Backbone signal assignments of 5F-Trp/U-^{15}N labeled 11Mut in the DRV-bound form, used to analyze the relaxation data in **Figure 3**.

		Chain A (ppm)				Chain B (ppm)				Not splitting (ppm)			
No.	AA	H	N	CA	CB	H	N	CA	CB	H	N	CA	CB
1	P				33.31								
2	Q	8.50	120.40	55.41	31.61								
3	I	9.52	128.70	60.55	40.38	9.52	128.69	60.55					
4	T									8.42	116.00	60.88	70.58
5	L												
6	W									7.28	120.62	58.82	
7	K									7.48	115.10	53.40	
8	R												
9	P												
10	L									7.70	126.60	53.80	
11	V	9.17	117.40	59.24	35.79	9.26	117.60	59.05	35.65				
12	T	8.45	117.90	62.85	69.91	8.51	118.30	62.97	69.17				
13	V									9.34	121.60	58.55	34.13
14	K	8.27	119.80	54.77	35.08	8.32	120.00	54.67	35.20				
15	I	9.25	125.90	58.11	40.51	9.14	125.60	58.36	40.63				
16	E	9.38	127.80	56.58	27.11	9.53	128.20	56.23	27.39				
17	G									8.75	103.20	45.53	
18	Q									8.09	120.50	54.44	30.69
19	L	8.60	125.10	54.73	42.34	8.57	124.70	54.63	42.33				
20	K	9.09	123.90	53.71	38.22	9.10	123.80	53.71	38.20				
21	E									8.76	121.20	55.44	31.70

		Chain 1 (ppm)				Chain 2 (ppm)				Not splitting (ppm)			
No.	AA	H	N	CA	CB	H	N	CA	CB	H	N	CA	CB
22	A	9.51	127.20	50.85	24.25	9.39	126.10	50.87	24.35				
23	L	8.92	121.90	53.24	45.83	8.62	120.50	53.64	45.76				
24	L	8.33	124.80	55.44	40.07	8.33	122.80	55.38	40.99				
25	D	8.55	128.50	52.18		8.70	127.30	53.11	43.33				
26	T	8.16	111.50	65.58	68.72	8.27	112.40	64.86	69.08				
27	G	8.01	108.90	45.16		9.18	108.60	44.95					
28	A	7.47	126.50	50.16	19.96	6.94	124.10	49.69	20.89				
29	D	8.69	125.20	57.42	41.08	8.14	123.80	57.79	41.56				
30	D	7.47	114.70	52.24	42.62	7.23	113.90	52.19	44.64				
31	T	8.18	116.90	63.08	71.06	7.97	118.40	63.92	71.68				
32	I	8.47	125.80	57.67	41.05	8.90	126.20	58.88	41.10				
33	F	9.25	127.50	55.76	43.12	9.38	126.70	56.01	44.24				
34	E	8.06	115.30	55.05	30.09	8.12	115.00	54.80	30.12				
35	E	7.81	116.40	58.57	29.82	7.46	116.20	58.70	29.91				

		Chain 1 (ppm)				Chain 2 (ppm)				Not splitting (ppm)			
No.	AA	H	N	CA	CB	H	N	CA	CB	H	N	CA	CB
36	M	7.01	120.40	54.61	33.82	6.96	120.40	54.58	34.74				
37	N	8.66	118.80	52.89	38.35	8.64	119.00	52.89	38.18				
38	L	7.12	124.00	51.80	41.97	7.29	124.10	51.65	42.69				
39	P												
40	G	8.32	105.50	44.42		8.27	105.00						
41	R									8.37	118.20	55.96	31.17
42	W	7.46	118.40	53.55	30.88	7.47	117.30	53.43	31.14				
43	K									8.40	118.60		
44	P												
45	I	8.50	119.10	59.73		8.49	120.30	59.52					
46	I	8.18	121.90	59.75	39.28	8.21	122.40	59.90	40.05				
47	I	8.90	123.50	58.89	42.13	8.79	123.70	59.23	40.78				
48	G	8.40	110.20	44.88		8.73	110.90	45.16					
49	G	7.38	105.60	43.61		7.68	108.80	44.81					
50	I												
51	G												
52	G	6.95	106.20	43.99		7.07	104.50	44.47					
53	F	8.32	117.60	57.17	41.20	8.31	118.60	56.56	41.86				
54	L	9.31	122.40	54.30	46.66	9.28	122.30	53.85	46.70				
55	K	8.48	125.30	56.68	32.30	8.51	126.30	57.08	32.17				
56	V	8.99	118.80	59.35	35.26	8.75	117.40	58.97	35.87				
57	R												
58	Q	9.67	123.10	55.96	32.18	9.78	123.60	56.21	32.16				
59	Y	9.40	128.90	57.55	40.97	9.37	128.90	57.79	41.28				
60	D	8.80	120.30	53.79	43.65	8.90	119.60	53.68	43.83				
61	Q									8.78	114.30	56.26	26.91
62	I									8.73	122.70	57.20	36.46
63	L	8.51	128.50	54.84	42.82	8.33	128.30	54.80	42.80				
64	I									9.13	127.00	59.92	41.25
65	E	8.75	125.80	54.49	31.47	8.84	126.20	54.47	31.45				
66	I									8.95	124.30	59.95	40.58
67	C									9.75	125.60	59.92	26.32
68	G									8.82	104.30	45.24	
69	H									8.20	118.80	54.38	29.81
70	K									8.94	125.10	56.86	32.76
71	V									9.09	122.50	59.79	34.69
72	I									8.24	122.10	58.89	40.10
73	G	8.94	113.80	45.77		8.99	113.60	45.77					
74	T	8.47	117.10	62.97	70.32	8.46	116.60	63.00	70.34				
75	V	8.88	126.20	61.38	36.73	8.67	123.90	61.41	36.79				

76	V	8.86	122.50	58.52	32.81	8.83	121.80	58.35	32.63				
77	V	9.01	120.90	59.39	35.80	9.06	118.80	58.65	35.82				
78	G	8.84	114.30	45.18		8.82	111.20	45.45					
79	P												
80	T	8.32	118.90	57.08	69.76	7.76	119.20	57.16	69.80				
80													
81	P												
82	F	7.08	112.40	56.26	43.67	7.06	115.30	57.15	43.44				
83	N	8.71	121.20	53.73	37.03	8.22	120.90	53.11	37.58				
84	V	9.34	124.70	61.34	35.59	9.32	122.10	61.20	?? 35.55				
85	I	8.89	124.10	58.88	35.50	9.28	124.60	58.96	35.64				
86	G	8.17	113.90	43.29		8.20	112.80	43.55					
87	R	9.34	116.40	61.46	30.03	9.09	116.40	61.30	29.85				
88	N	7.99	115.10	56.91	36.32	7.94	114.90	56.73	36.66				
89	L									7.05	116.40	55.68	52.26
90	L									8.24	57.90	57.88	41.97
91	T	7.91	106.50	63.66	68.13	7.87	106.40	63.66	68.15				
92	Q									6.78	119.40	57.36	29.44
93	I	6.88	109.50	60.71	37.65	6.86	109.10	60.63	37.65				
94	G	7.17	107.80	46.03		7.16	107.40	46.06					
95	C	7.28	117.20	58.68	28.15	7.32	117.60	58.40	28.05				
96	T	9.22	116.30	59.61	72.40	9.21	117.20	59.66	72.40				
97	L	8.45	120.00	53.51	45.51	8.52	119.90	53.44	45.44				
98	N									8.86	121.00	53.02	41.98
99	F									8.00	121.90	58.80	38.63

Footnote: See **Table S2** footnote; backbone connectivities were obtained via HNCACB experiment. Filtered NOESY data are not available for 11Mut/DRV. Thus, most backbone signals were tentatively traced to either Chain 1 or 2 to make the chain assignments consistent to those of wt-PR/DRV. This assignment is sufficient only for relative comparison of relaxation data between wt-PR/DRV and 11Mut/DRV.

Table S4. ^{19}F chemical shifts, intensities and areas of 5F-Trp resonances of wt-PR, 4Mut, 10Mut and 11Mut in solution.[1]

WT	CS (ppm)	Intensity[1]	Int_error	Area[1]	Area_error
W6	-47.81	0.55	0.011	0.45	0.016
W42	-49.32	0.45	0.009	0.55	0.020
**4Mut**	**CS (ppm)**	**Intensity**	**Int_error**	**Area**	**Area_error**
W6	-47.75	0.55	0.012	0.49	0.019
W42	-48.69	0.45	0.011	0.51	0.023
**10Mut**	**CS (ppm)**	**Intensity**	**Int_error**	**Area**	**Area_error**
W6	-47.75	0.52	0.011	0.43	0.017
W42	-48.64	0.48	0.010	0.57	0.020
**11Mut**	**CS (ppm)**	**Intensity**	**Int_error**	**Area**	**Area_error**
W6	-47.75	0.51	0.007	0.42	0.010
W42	-48.57	0.49	0.006	0.58	0.013

[1] Intensities and areas were determined using the line-shape analysis feature of Bruker Topspin 4.4.0 (Bruker, Billerica, MA). To compare relative peak intensity, the cumulative height of the listed peaks in each protein was normalized to 1.0. Similarly, the cumulative peak area of the listed peaks in each protein was normalized to 1.0. Area error was calculated from the intensity and width using the error propagation equation.

Table S5. ^{19}F chemical shifts, intensities and areas of 5F-Trp resonances of wt-PR, 4Mut, 10Mut and 11Mut in the DRV-bound forms in solution.[1]

WT	CS (ppm)	Intensity[2]	Int_error	Area[2]	Area_error
W6a	-47.62	0.33	0.005	0.30	0.009
W6b	-47.73	0.26	0.006	0.15	0.008
W42a	-49.25	0.41	0.003	0.55	0.008
W42b	-	-	-	-	-
**4Mut**	**CS (ppm)**	**Intensity**	**Int_error**	**Area**	**Area_error**
W6a	-47.58	0.34	0.008	0.37	0.018
W6b	-47.72	0.25	0.012	0.14	0.014
W42a	-48.71	0.41	0.006	0.49	0.013
W42b	-	-	-	-	-
**10Mut**	**CS (ppm)**	**Intensity**	**Int_error**	**Area**	**Area_error**
W6a	-47.73	0.48	0.006	0.46	0.011
W6b	-	-	-	-	-
W42a	-48.60	0.25	0.006	0.23	0.011
W42b	-48.90	0.28	0.006	0.32	0.013
**11Mut**	**CS (ppm)**	**Intensity**	**Int_error**	**Area**	**Area_error**
W6a	-47.72	0.48	0.008	0.49	0.014
W6b	-	-	-	-	-
W42a	-48.60	0.25	0.009	0.20	0.014
W42b	-48.92	0.27	0.007	0.32	0.016

[1] Resonances of W6 were tentatively named as W6a and W6b, for downfield and upfield resonances, respectively, when they split to two. Similarly, resonances of W42 were tentatively named as W42a and W42b, for downfield and upfield resonances, respectively, when they split to two.

[2] Intensities and areas were determined using the line-shape analysis feature of Bruker Topspin 4.4.0 (Bruker, Billerica, MA). To compare relative peak intensity, the cumulative height of the listed peaks in each protein was normalized to 1.0. Similarly, the cumulative peak area of the listed peaks in each protein was normalized to 1.0. Area error was calculated from the intensity and width using the error propagation equation.

Table S6, Temperature-dependence of ^{19}F chemical shifts and resonance intensities of 11Mut in the DRV-bound form in solution[1].

283K	CS (ppm)	Intensity	Int_error	Area	Area_error
W6	-47.80	0.51	0.008	0.50	0.013
W42a	-48.71	0.23	0.007	0.24	0.017
W42b	-49.08	0.26	0.007	0.26	0.015
**293K**	**CS (ppm)**	**Intensity**	**Int_error**	**Area**	**Area_error**
W6	-47.70	0.47	0.008	0.47	0.014
W42a	-48.58	0.26	0.008	0.25	0.017
W42b	-48.91	0.28	0.008	0.28	0.016
**298K**	**CS (ppm)**	**Intensity**	**Int_error**	**Area**	**Area_error**
W6	-47.66	0.46	0.008	0.46	0.014
W42a	-48.54	0.26	0.008	0.25	0.018
W42b	-48.84	0.28	0.008	0.29	0.016
**305K**	**CS (ppm)**	**Intensity**	**Int_error**	**Area**	**Area_error**
W6	-47.59	0.45	0.008	0.45	0.015
W42a	-48.47	0.27	0.009	0.25	0.018
W42b	-48.74	0.28	0.008	0.30	0.017

[1] Resonances of W42 were tentatively named as W42a and W42b, for downfield and upfield resonances, respectively, when they split to two.

[2] Intensities and areas were determined using the line-shape analysis feature of Bruker Topspin 4.4.0 (Bruker, Billerica, MA). To compare relative peak intensity, the cumulative height of the listed peaks in each protein was normalized to 1.0. Similarly, the cumulative peak area of the listed peaks in each protein was normalized to 1.0. Area error was calculated from the intensity and width using the error propagation equation.

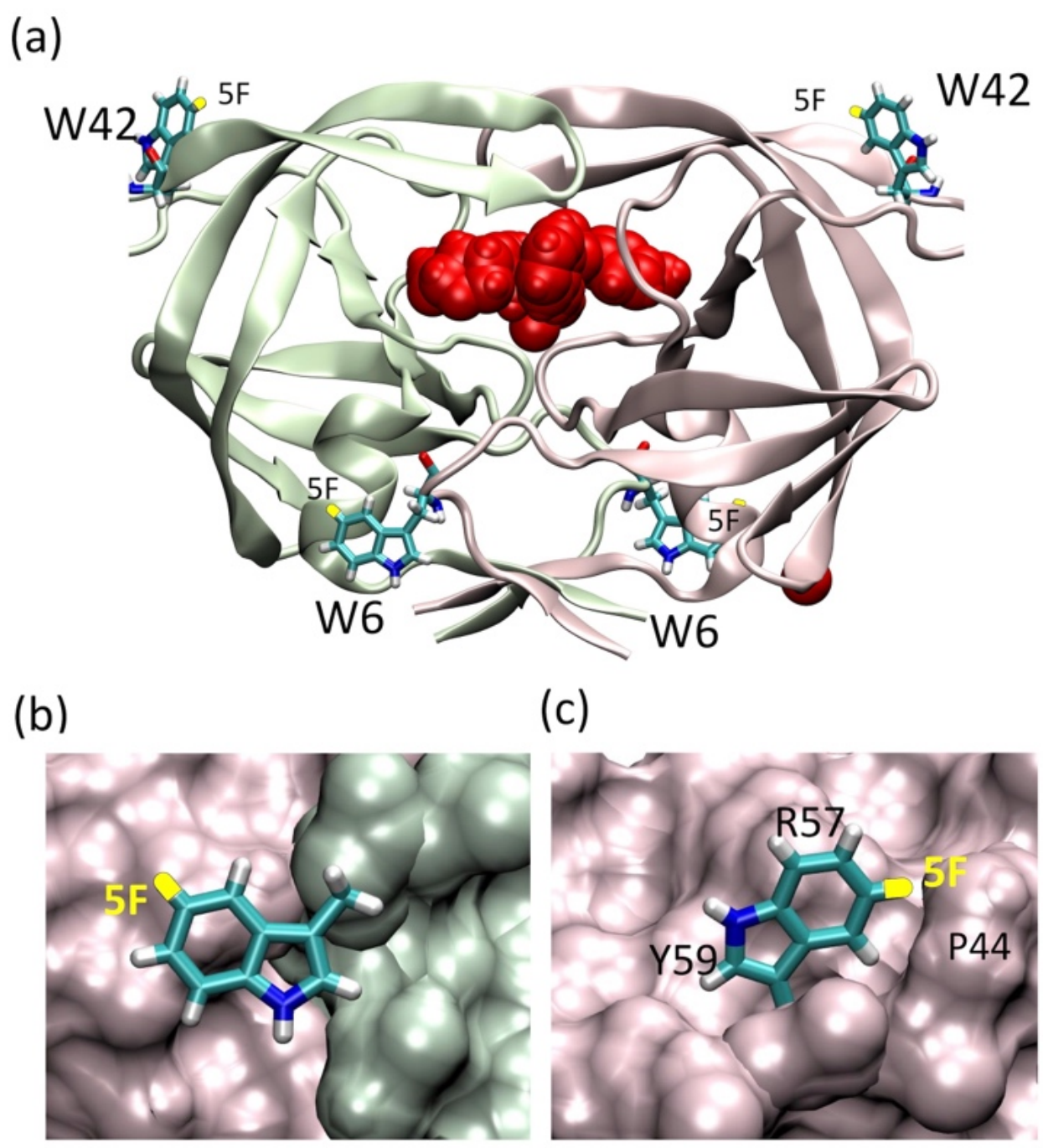

Figure S1. Cartoon of (a) HIV-1 wt-PR showing positions of (b) W6 and (c) W42. In all panels, tryptophans are shown by sticks, and the position 5 is marked with yellow mark. W6 indole is exposed to solution (panel (b)). W42 indole is partially buried in the protein core with R57 and Y59 in one side of the ring and P44 the other side.

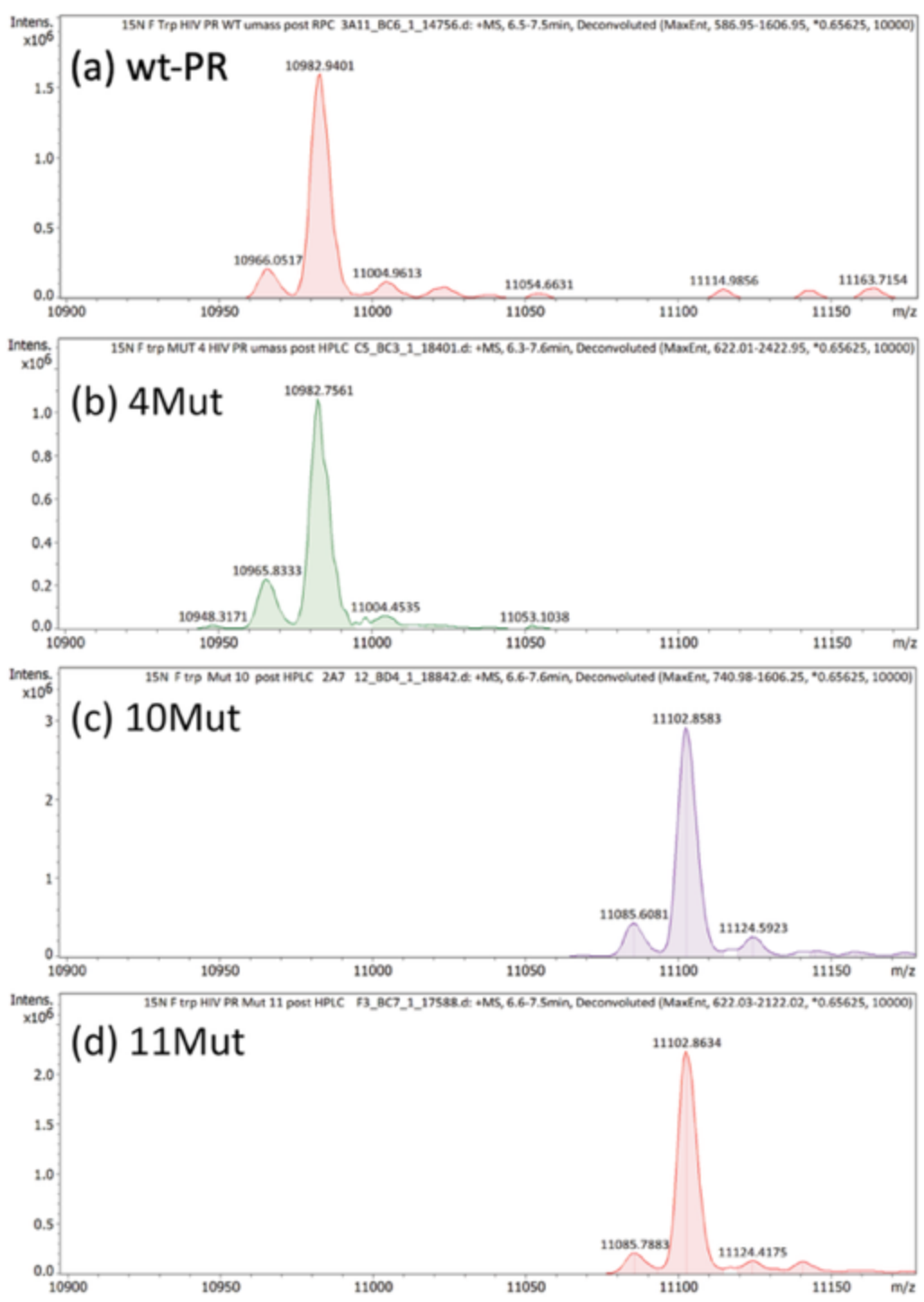

Figure S2. Typical mass fractions of 5F-Trp/U-^{15}N labeled wt-PR, 4Mut, 10Mut and 11Mut, showing 10-15% of the total having ^{19}F-labeling at only one Trp.

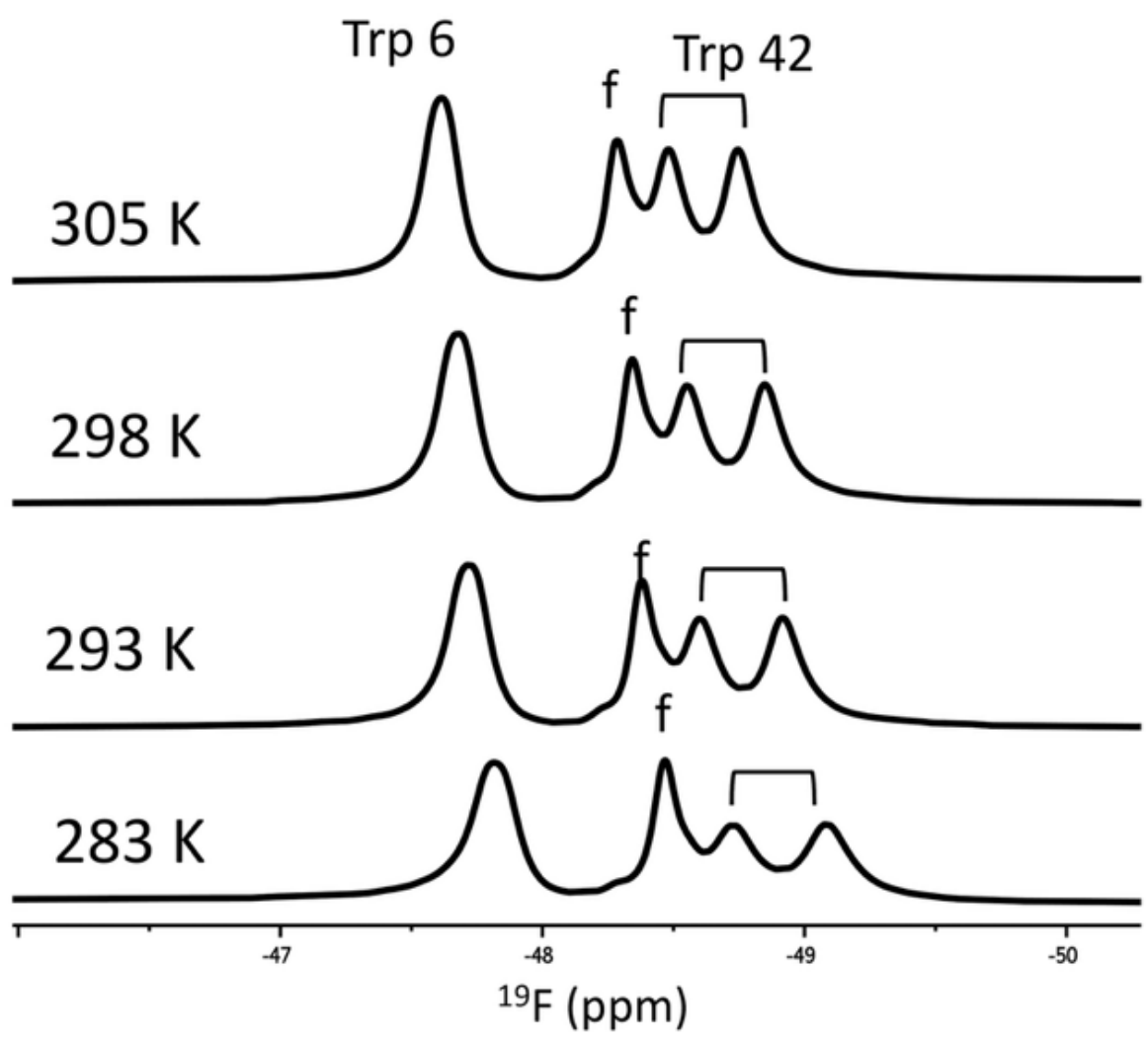

Figure S3. Temperature dependence of 11Mut in the presence of DRV in solution. “f” indicates fragment resonance. Note, chemical shift reference was adjusted at 293 K spectrum only.

REFERENCES

Henes, M., Lockbaum, G.J., Kosovrasti, K., Leidner, F., Nachum, G.S., Nalivaika, E.A., Lee, S.K., Spielvogel, E., Zhou, S., Swanstrom, R., Bolon, D.N.A., Kurt Yilmaz, N., Schiffer, C.A. (2019) Picomolar to Micromolar: Elucidating the Role of Distal Mutations in HIV-1 Protease in Conferring Drug Resistance. ACS Chem Biol 14, 2441-2452.

Khan, S.N., Persons, J.D., Guerrero, M., Ilina, T.V., Oda, M., Ishima, R. (2021) A synergy of activity, stability, and inhibitor-interaction of HIV-1 protease mutants evolved under drug-pressure. Protein Sci 30, 571-582.

Khan, S.N., Persons, J.D., Paulsen, J.L., Guerrero, M., Schiffer, C.A., Kurt-Yilmaz, N., Ishima, R. (2018) Probing Structural Changes among Analogous Inhibitor-Bound Forms of HIV-1 Protease and a Drug-Resistant Mutant in Solution by Nuclear Magnetic Resonance. Biochemistry 57, 1652-1662.

Paulsen, J.L., Leidner, F., Ragland, D.A., Kurt Yilmaz, N., Schiffer, C.A. (2017) Interdependence of Inhibitor Recognition in HIV-1 Protease. J Chem Theory Comput 13, 2300-2309.

Persons, J.D., Khan, S.N., Ishima, R. (2018) An NMR strategy to detect conformational differences in a protein complexed with highly analogous inhibitors in solution. Methods 148, 9-18.
